# Flavonoid-Conjugated Gadolinium Complexes as Anti-Inflammatory Theranostic Agents

**DOI:** 10.3390/antiox11122470

**Published:** 2022-12-15

**Authors:** Byeong Woo Yang, Sohyeon Yang, Soyeon Kim, Ah Rum Baek, Bokyung Sung, Yeoun-Hee Kim, Jung Tae Lee, Sang Yun Lee, Hee-Kyung Kim, Garam Choi, Ji-Ae Park, Sung-Wook Nam, Gang-Ho Lee, Yongmin Chang

**Affiliations:** 1 Department of Medical & Biological Engineering, Kyungpook National University, 80 Daehak-ro, Buk-gu, Daegu 41566, Republic of Korea; 2 Department of Medical Science, School of Medicine, Kyungpook National University, 680 Gukchaebosang-ro, Jung-gu, Daegu 41944, Republic of Korea; 3 Division of Applied RI, Korea Institute of Radiological & Medical Sciences (KIRAMS), 75 Nowon-ro, Nowon-gu, Seoul 01812, Republic of Korea; 4 Institute of Biomedical Engineering Research, Kyungpook National University, 680 Gukchaebosang-ro, Jung-gu, Daegu 41944, Republic of Korea; 5 R&D Center, Etnova Therapeutics Corp., 124, Sagimakgol-ro, Jungwon-gu, Seongnam-si 13207, Republic of Korea; 6 Preclinical Research Center, Daegu-Gyeongbuk Medical Innovation Foundation, 88 Dongnae-ro, Dong-gu, Daegu 41061, Republic of Korea; 7 Department of Molecular Medicine, School of Medicine, Kyungpook National University, 680 Gukchaebosang-ro, Jung-gu, Daegu 41944, Republic of Korea; 8 Department of Chemistry, Kyungpook National University, 80, Daehak-ro, Buk-gu, Daegu 41566, Republic of Korea; 9 Department of Radiology, Kyungpook National University Hospital, 130 Dongdeok-ro, Jung-gu, Daegu 41944, Republic of Korea

**Keywords:** phytochemical, flavonoid, inflammation, gadolinium complex, theranostics

## Abstract

In this study, we designed, synthesized, and evaluated gadolinium compounds conjugated with flavonoids as potential theranostic agents for the treatment of inflammation. These novel theranostic agents combine a molecular imaging agent and one of three flavonoids (galangin, chrysin, and 7-hydroxyflavone) as anti-inflammatory drugs as a single integrated platform. Using these agents, MR imaging showed contrast enhancement (>10 in CNR) at inflamed sites in an animal inflammation model, and subsequent MR imaging used to monitor the therapeutic efficacy of these integrated agents revealed changes in inflamed regions. The anti-inflammatory effects of these agents were demonstrated both in vitro and in vivo. Furthermore, the antioxidant efficacy of the agents was evaluated by measuring their reactive oxygen species scavenging properties. For example, Gd-galangin at 30 μM showed a three-fold higher ROS scavenging of DPPH. Taken together, our findings provide convincing evidence to indicate that flavonoid-conjugated gadolinium compounds can be used as potentially efficient theranostic agents for the treatment of inflammation.

## 1. Introduction

Flavonoids, a class of phytochemicals found in high quantities in vegetables, fruits, tea, and red wine, are known to have strong antioxidant potency via the direct scavenging of reactive oxygen species (ROS), upregulation of ROS-eliminating enzymes, and downregulation of inducible nitric oxide synthase (iNOS). ROS plays key roles in the induction of inflammation via the release of inflammatory signal molecules and the activation of macrophages to promote the release of IL-6. Flavonoids have been shown to inhibit proinflammatory transcription factors such as NF-κB, thereby suppressing the synthesis and release of inflammatory TNF-α, IL-6, and iNOS [1,2]. Among the described flavonoids, the molecules of galangin (3,5,7-trihydroxyflavone), chrysin, and flavone contain three, two, and one hydroxyl groups, respectively, and are derived primarily from *Zingiber officinale* (ginger), *Alpinia officinarum*, and *Helichrysum aureonitens*. Previous studies have reported on the beneficial properties of these polyphenolic compounds, including their antioxidant, antitumor, anti-inflammatory, antimicrobial, and antiviral activities [3,4,5,6,7].

Theranostics is often defined as a therapeutic approach that combines therapy and diagnostic imaging. In contrast to the use of separate agents for therapy and imaging, theranostic agents combine these features in a single agent, which confers the ability to overcome differences in biodistribution and selectivity that exist between distinct imaging and therapeutic agents [8,9,10,11]. Theranostic agents are thus characterized by their ability to image and monitor disease progression, delivery kinetics, and drug efficacy, along with presenting the opportunity to fine-tune therapy toward more effective and personalized medicine. Given that theranostic agents can simultaneously deliver therapeutic drugs and diagnostic imaging agents within the same dose, the effective dose for therapeutic drugs and imaging agents should be considered to optimize the efficacy of these agents.

When administering flavonoids as therapeutic drugs, it is necessary to use micromolar concentrations, as concentrations within the 10–100 μM range are typically required for in vivo antioxidant activity, which is one or two orders higher than the concentrations in plasma [12,13]. Therefore, for theranostic purposes, gadolinium compounds, which are low-molecular weight magnetic resonance imaging (MRI) agents, are considered among the most suitable for use in combinations with flavonoids. The clinical dose of gadolinium compounds is similarly in the range between 10 and 100 μM, and thus is well-matched for use in combination with flavonoids. Furthermore, as highly water-soluble agents, gadolinium compounds can enhance the solubility of flavonoids, which is an important consideration, as the solubility of drug candidates, including theranostic agents, is among the pivotal physicochemical properties of drugs [14]. Indeed, candidate drugs are commonly required to have solubilities of approximately 10 µM for preclinical evaluation [15]. Consequently, gadolinium compounds are considered good candidates for enhancing the solubility of flavonoids to optimize theranostic agents as promising drug candidates.

In this study, we sought to design, synthesize, and evaluate inflammation-targeted gadolinium compounds conjugated with flavonoids as potential theranostic agents for the treatment of inflammation. These novel agents combine a molecular imaging agent and a flavonoid as anti-inflammatory drugs within a single integrated platform, using which, MR imaging can be undertaken to detect inflamed areas at the molecular level in animal models of lipopolysaccharide (LPS)-induced inflammation. Furthermore, subsequent MR imaging can be employed to monitor the therapeutic efficacy of these integrated agents by identifying changes in the inflamed areas. We also examined the potential antioxidant activity of the synthesized theranostic agents to determine the antioxidant efficacy of agents combining flavonoids and imaging compounds.

## 2. Materials and Methods

### 2.1. Materials

Galangin (3,5,7-Trihydroxyflavone) was purchased from Aktin Chemicals, Inc. (Chengdu, China), and tri-tert-butyl 1,4,7,10-tetraazacyclododecane-1,4,7-triacetate hydrobromide was purchased from Angene International Ltd. (Nanjing, China). The isolation of pure products was carried out via silica gel flash chromatography. Silica gel (particle size: 60–200 μm) was purchased from Intertechnologies Co., Ltd. (Seoul, Republic of Korea). Lipopolysaccharides derived from *Escherichia coli* (O127:B8) were purchased from Sigma Aldrich (St. Louis, MO, USA). Other reagents and solvents were purchased from Sigma Aldrich, Tokyo Chemical Industry Co., Ltd. (Tokyo, Japan) and Ducksan Pure Chemicals Co., Ltd. (Ansan-si, Gyeonggi-do, Republic of Korea).

### 2.2. Instrument

^1^H NMR (500 Hz) and ^13^C NMR (125 Hz) spectra were recorded using Bruker Advance 500 nuclear magnetic resonance (NMR) spectrometer. Melting point measurements were performed using an MPA100 automated melting point device (Stanford Research Inc., Sunnyvale, CA, USA). High-resolution fast atom bombardment mass spectrometry (HR-FAB-MS) was performed using a JMS-700 model spectrometer (Jeol, Tokyo, Japan) at the Korea Basic Science Center, Daegu, Republic of Korea. A high-performance liquid chromatography system (prep-HPLC; LC/Forte/R, YMC, Kyoto, Japan) and a YMC-Hydrosphere C18 column (250 × 20.0 mm. inner diameter, particle size: 5 μm, pore size: 12 nm, YMC) were utilized for purification. The prep-HPLC system used ultraviolet-visible (UV-vis) detection at 254 and 320 nm. Based on the HPLC spectra, the purity of products was assessed to be greater than 95%. A flash column chromatography system (Isolera Prime, Biotage, Uppsala, Sweden) incorporating a SNAP KP-C18-HS 120 g cartridge was used for purification. The system used UV-vis detection at 254 and 320 nm. Gd ion concentration data used to confirm lipophilicity were obtained using Optima 7300DV and Avio 500 inductively coupled plasma (ICP) spectrometers (Perkin Elmer, Waltham, MA, USA), and UV-vis absorption and fluorescence measurements were taken using a SpectraMax^®^ i3 microplate reader (Molecular Devices, San Jose, CA, USA) in conjunction with 96-well cell culture plates at 25 °C.

### 2.3. Synthesis and Characterization

A schematic diagram of the synthesis of the gadolinium complex comprising DO3A conjugated with 7-hydroxyflavone, chrysin, and galangin is presented in Figure 1. The supplementary material summarizes detailed procedures for synthesis, and includes ^1^H NMR, ^13^C NMR, HR-FAB-MS, and HPLC spectra as characterized data (Figures S1–S32).

### 2.4. Relaxivity

Samples used for relaxivity determinations were prepared at five concentrations (0.0625, 0.125, 0.25, 0.5, and 1 mM) in deionized water, phosphate-buffered saline (PBS), or 0.67 mM human serum albumin (HSA) solution [16]. The three synthesized Gd complexes and various commercial contrast agents Gd-BT-DO3A (Gadovist^®^), Gd-DTPA-EOB (Primovist^®^), Gd-DTPA-BOPTA (MultiHance^®^), Gd-DTPA (Magnevist^®^), and Gd-DOTA (Dotarem^®^)) were measured together using a Signa Architect 3.0 T system (128 MHz, GE Healthcare, Milwaukee, WI, USA).

*T*_1_ relaxivity (*r*_1_) was measured using the inversion recovery technique, with several inversion times (TI) ranging from 50 to 1750 ms. *T*_1_ relaxation times were estimated using the nonlinear least-squares fit of the measured signal intensity at each TI value. *T*_2_ relaxivity (*r*_2_) was measured using multiple spin-echo technique with a Carr-Purcell-Meiboon-Grill pulse sequence. *T*_2_ relaxation times were estimated using the nonlinear least-squares fit of the measured signal intensity at each TE. *r*_1_ and *r*_2_ were calculated using a linear fit at each relaxation rate and concentration of the Gd complex solution. Relaxation times (*T*_1_ and *T*_2_) map and relaxivity (*r*_1_ and *r*_2_) map were also obtained.

### 2.5. Octanol-Water Partition Coefficients

Octanol-water partition coefficient experiments to determine lipophilicity were performed using a previously described method [17]. Samples were prepared by mixing equal volumes (1 mL) of 1-octanol and 1 mM solution of Gd complex dissolved in deionized water. The immobilized samples were shaken vigorously on a rotator for 48 h and then allowed to stand at room temperature for 24 h. Samples collected from the separated water and octanol layers were pretreated by digestion with hydrochloric acid (35%; Ducksan, Ansan-si, Gyeonggi-do, Republic of Korea) and nitric acid (70%; Ducksan, Ansan-si, Gyeonggi-do, Republic of Korea) at high temperature. Ga^3+^ ion concentrations were determined using ICP-MS and octanol-water partition coefficients were calculated using Equation (1) as follows:

(1) $$\log P=\log\frac{C_{o}}{C_{w}}$$

where *log P* is the logarithm of the partition coefficient, *Co* is the Gd^3+^ concentration of the 1-octanol layer, and *C_w_* is the Gd^3+^ concentration of the water layer.

### 2.6. Stability Measurement: Transmetalation Kinetics and pH Stability

The measurement of transmetalation kinetics has been described previously [18,19]. Briefly, transmetalation can be estimated through the evolution of the transverse relaxation rate. In phosphate buffer, diamagnetic Zn^2+^ ions promote transmetalation of the Gd complexes, and the released Gd^3+^ ions combine with PO_4_^3−^ ions in solution to form GdPO_4_. A 10 μL volume of 250 mM ZnCl_2_ was added to 1 mL of phosphate-buffered solution containing 2.5 mM Gd complex. As control groups, we compared this preparation with the commercial contrast agents mentioned in Section 2.4. Relaxivity. Measurements were taken using the Signa Architect 3.0 T system for 72 h at room temperature. The relaxation rate is expressed as (*R*_2_(*t*)/*R*_2_(0)).

For pH stability experiments [18], samples were prepared at the same concentration in buffers with different pH values (pH 1, 3, 5, 7, 9, and 11). The synthesized Gd complexes and commercial MR contrast agents were compared for 7 days.

### 2.7. LPS-Induced Inflammatory Animal Models

The mice used in this study were BALB/c mice (7–8 weeks old, weight: 22–25 g) obtained from HanaBio (Pyeongtaek-si, Gyeonggi-do, Republic of Korea). The mice were maintained in cages at 21 ± 1 °C, on a 12-h light/dark cycle, with water and food provided ad libitum throughout the experiments. All studies using animals were conducted in accordance with the guidelines of the Animal Protection and Use Committee of Kyungpook National University (2022-0345). Mice were anesthetized with 1.5–2.0% isoflurane in oxygen, and to induce inflammation, the left thighs of mice were shaved and LPS (5 mg/kg body weight, 40 μL) was injected intramuscularly [20]. At 12 h after LPS injection, Gd-galangin was injected intravenously into the tail vein, and the mice were subsequently euthanized for tissue collection at 24 h after LPS injection.

For the in vivo measurement, mice were divided into the following four groups (*n* = 6 for each group):

Group I: Normal

Group II: LPS

Group III: LPS and Gd-galangin 0.05 mmol/kg 

Group IV: LPS and Gd-galangin 0.1 mmol/kg 

Mice were perfused with saline and the muscle tissues were extracted.

### 2.8. In Vivo MR Imaging

In vivo MR imaging was performed using a 3.0 T unit (Magnetom Tim Trio; Siemens, Erlangen, Germany) equipped with a six-channel rat body coil. The mice were anesthetized with 1.5–2.0% isoflurane in oxygen during MR scanning. At 24 h after LPS injection, Gd-galangin and Gd-BT-DO3A at a concentration of 0.1 mmol Gd/kg were injected intravenously into the tail vein.

The coronal imaging parameters used were as follows: repetition time (TR) = 385, echo time (TE) = 10, field of view (FOV) = 70 mm, matrix size = 192 × 134, slice thickness = 1.0 mm, number of excitations (NEX) = 8 and scan time = 3 min 28 s. The axial imaging parameters used were as follows: TR = 385, TE = 10, FOV = 70 mm, matrix size = 192 × 134, slice thickness = 1.0 mm, NEX = 4, and scan time = 1 min 45 s. ImageJ software (1.53 k; NIH, Bethesda, MD, USA) was used for MR image analysis. The signal intensities (SIs) of the heart, liver, gallbladder, kidneys, and inflamed tissue were measured by drawing regions of interest (ROI). The signal-to-noise ratio (SNR) of the ROIs was calculated by dividing the corresponding SI by the noise. Using the SNR, the contrast-to-noise ratio (CNR) was calculated using the following Equation (2):

(2) $$\mathrm{CNR}=\frac{{\mathrm{SI}}_{\mathrm{post}}}{{\mathrm{Noise}}_{\mathrm{post}}}-\frac{{\mathrm{SI}}_{\mathrm{pre}}}{{\mathrm{Noise}}_{\mathrm{pre}}}$$

To determine the contrast between normal and inflamed tissues, we calculated CNR_inflamed tissue_ using the following Equation (3):

(3) $${\mathrm{CNR}}_{\mathrm{inflamed}\;\mathrm{tissue}}=\frac{{\mathrm{SI}}_{\mathrm{inflamed}\;\mathrm{tissue}}}{{\mathrm{Noise}}_{\mathrm{inflamed}\;\mathrm{tissue}}}-\frac{{\mathrm{SI}}_{\mathrm{normal}\;\mathrm{tissue}}}{{\mathrm{Noise}}_{\mathrm{normal}\;\mathrm{tissue}}}$$

### 2.9. Biodistribution

BALB/c mice (6–7 weeks old, weight: 20–22 g) were injected intravenously with 0.1 mmol Gd/kg in the tail vein. At different time points post-injection (15 min, 30 min, 1 h, 3 h, and 24 h), the mice were sacrificed by exsanguination from the vena cava. Organs (brain, heart, liver, kidney, spleen, gallbladder, and intestines) were harvested and digested with nitric acid (70%; Ducksan, Ansan-si, Gyeonggi-do, Republic of Korea) and hydrogen peroxide (30%; Ducksan, Ansan-si, Gyeonggi-do, Republic of Korea) at 180 °C for 2 h. All samples were prepared by diluting in 3% nitric acid after filtering. The Gd^3+^ concentrations of diluted sample solutions were measured using ICP-AES [21].

### 2.10. In Vivo IVIS Imaging

Mice were anesthetized with 1.5–2.0% isoflurane in oxygen. To induce inflammation, the left thighs of the mice were shaved and LPS (5 mg/kg body weight, 40 μL) was injected intramuscularly. At 24 h after LPS injection, the luminol derivative L-012 (Wako Pure Chemical Industries, Ltd., Osaka, Japan) was injected intramuscularly, and 20 min later, luminescence imaging was performed to confirm the generation of ROS. At 1 h after first L-012 injection, mice were intravenously injected with saline or Gd-galangin, and having subsequently been administered L-012, luminescence images were obtained to assess the ROS scavenging effect (Figure 5a).

### 2.11. Cell Culture

For in vitro studies, we used the RAW 264.7 murine macrophage cell line (ATCC^®^TIB-71) purchased from the American Type Culture Collection (ATCC, Manassas, VA, USA). Cells were also obtained from the Korean Cell Line Bank (KCLB, Seoul, Republic of Korea). These cells were cultured in Dulbecco’s modified Eagle’s medium (DMEM; WELGENE, Daegu, Republic of Korea) supplemented with 10% filtered fetal bovine serum (FBS, Cat. SH3099.03; Hyclone, UT, USA) and 1% penicillin-streptomycin (PS; Gibco, MA, USA). Cells were maintained at 37 °C in a 5% CO_2_ humidified incubator.

### 2.12. Cell Viability Assay

Cell viability was evaluated using a D-Plus™ CCK cell viability assay kit (Cat. CCK-300; Dongin LS, Daegu, Republic of Korea). RAW 264.7 macrophage cells were seeded into 96-well plates at 5 × 10^4^ cells/well and left to 24 h to settle. The cells were subsequently treated with 1, 5, 10, 25, 50, 75, or 100 μM of Gd-galangin in a serum-free medium for a further 24 h [22]. Thereafter, 10 μL of CCK reagent was added to each well, followed by incubation for 1 h at 37 °C. The absorbance of samples was measured at 450 nm using a SpectraMaxi3 microplate reader. The experiment was performed independently more than three times.

### 2.13. Cell Fractionation

To confirm the translocation of NF-κB from the cytoplasm to the nucleus, we used a NE-PER kit (Nuclear and Cytoplasm Extraction Reagents, Cat. 78835; Thermo Fisher Scientific, Waltham, MA, USA) to extract the cytosol and nuclei from whole RAW 264.7 cells in accordance with the manufacturer’s instructions [23], with subsequent quantification using a Pierce BCA Protein Assay Kit (Thermo Fisher Scientific).

### 2.14. Reactive Oxygen Species Measurement

RAW 264.7 cells were seeded in two types of plate. To obtain microscopic images of the cells, 5 × 10^5^ cells were seeded in 35-mm black confocal dishes. Cells were also seeded in black 96-well plates (5 × 10^4^ cells/well) to measure fluorescence intensity. Cells were activated with LPS for 1 h, after which the medium was replaced with fresh serum-free medium containing two different concentrations of Gd-galangin (25 or 50 μM). After 4 h, cells were washed out with Hank’s balanced salt solution (HBSS; WELGENE, Daegu, Republic of Korea), followed by the addition of 20 μM of H_2_DCFDA dye (Cat. D399; Thermo Fisher Scientific) in serum-free medium and incubation for 45 min in the dark. Thereafter, the wells of the black plates were again washed with HBSS, followed by the addition of 100 μL of HBSS before measurements. Fluorescence intensity was measured at excitation and emission wavelengths of 480 and 530 nm, respectively, at the endpoint of the experimental time.

Medium in the black confocal dishes was also replaced with HBSS and cells were fixed with 4% paraformaldehyde for 10 min. Thereafter, the dishes were washed out with three 5 min washes using Tris-buffered saline pH 7.6. Confocal regions were mounted with Vectashield antifade mounting medium (Vectashield H-1000; Vector Laboratories, Inc. Burlingame, CA, USA). Images were obtained using a Nikon fluorescence microscope (ECLIPSE Ti; Nikon, Tokyo, Japan), and mean fluorescence intensity values were determined using NIS-Elements BR 5.11 software (Nikon, Tokyo, Japan). The experiment was performed independently more than three times.

### 2.15. Nitric Oxide Assay

To determine levels of nitric oxide (NO), we used a Griess Reagent Kit (Cat. G7921; Invitrogen, Carlsbad, CA, USA). RAW 264.7 cells were seeded in 6-well plates (1 × 10^6^ cells/well) and left to settle. At 2 h after stimulation with LPS, the cells were treated with one of three different concentrations of Gd-galangin (10, 25, or 50 μM), and following a 12 h incubation, the medium was collected and briefly centrifuged at 5,000 rpm. The resulting supernatants were collected and used to measure nitrite oxide production. The assay was performed according to the manufacturer’s instructions [24], with absorbance being measured at 540 nm using a SpectraMaxi3 microplate reader. The experiment was performed independently at least three times.

### 2.16. Western Blot Analysis

The tissues or whole cells were lysed with radio-immunoprecipitation assay lysis buffer (RIPA buffer; Millipore, Bedford, MA, USA) containing a protease and phosphatase inhibitor cocktail (Cat. 04 906 837 001, Roche Diagnostics, Basel, Switzerland) for 1 h. Lysates were centrifuged at 13,000 rpm at 4 °C and homogenates were collected carefully not to take any pellets or remaining fat.

All of the samples were quantified with Pierce BCA Protein Assay Kit (Thermo Fisher Scientific) [25]. The same amounts of protein were loaded and migrated with SDS-PAGE (Bio-Rad Laboratories Inc., Hercules, CA, USA) and the following antibodies were used for our study: β-actin (Cat. sc-47778) was purchased from Santa Cruz Biotechnology, Lamin B1 (Cat. 12586), iNOS (Cat. 13120), ASC (Cat. 67824), NF-κB (Cat. 6956), IκBα (Cat. 4814), pIκBα (Cat. 2859), pp38 (Cat. 9215), p38 (Cat. 9212), pJNK (Cat. 9251), JNK (Cat. 9252), pErk (Cat. 9101) and Erk (Cat. 9102) were purchased from Cell Signaling Technologies, IL-1β (Cat. P420B), Nrf2 (Cat. PA5-88084), pNrf2 (Cat. PA5-67520) and HO-1 (Cat. PA5-77833) were purchased from Invitrogen, and NLRP3 (Cat. ab270449) was purchased from Abcam. Horseradish peroxidase (HRP)-conjugated secondary antibodies (Cell Signaling Technologies, Danvers, MA, USA) were diluted to 1:2000 and incubated for 1 h at room temperature. The protein bands were enhanced with chemiluminescence (ECL) solution and captured using the Chemiluminescence Western Imaging System (Supernova-Q1800TM, Centronics, Daejeon, Republic of Korea and Amersham ImageQuant 800, Cytiva, Marlborough, MA, USA).

Densitometric values were obtained using ImageJ software (Version 1.53q; US National Institutes of Health, Bethesda, MD, USA) and data were normalized to β-actin, Lamin B1, or the total form of phosphorylated factors. Full bands for all western blots were included in Figure S36.

### 2.17. Statistical Analysis

All data were analyzed and presented using GraphPad Prism version 5.03 (GraphPad Prism software Inc., San Diego, CA, USA), and values are shown as the means ± SEM (standard error of the mean). The data were evaluated using unpaired two-tailed *t*-tests with *p*-values < 0.05 considered to be statistically significant.

## 3. Results

### 3.1. Synthesis

**Figure 1:**
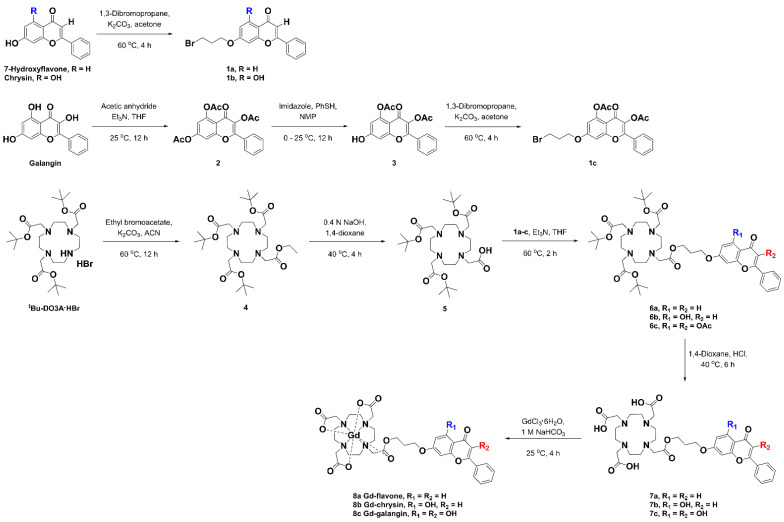
Synthesis of the gadolinium complexes of the DO3A conjugated with 7-hydroxyflavone, chrysin and galangin. Abbreviations: triethylamine (Et_3_N), tetrahydrofuran (THF), thiophenol (PhSH), 1-methyl-2-pyrrolidone (NMP), acetonitrile (ACN).

The hydroxyl group at position seven of the flavonoid ring A is more acidic than other positions [26,27]. The hydroxyl group at position seven with high reactivity synthesized **1a**–**c** bonded to 1,3-dibromopropane as a linker. Galangin intermediates protected by acetyl group (**2**) reacted with imidazole and thiophenol (PhSH) to allow the linker to react at the same position (**3**) [28,29]. After synthesizing *^t^*Bu-DO3A-COOH (**5**) according to the literature, intermediate **6** formed an ester bond with **1a**–**c** under triethylamine and tetrahydrofuran (THF) conditions. Following deprotection of the *tert*-butyl and acetyl groups with hydrochloric acid (**7a**–**c**), the gadolinium complexes (**8a** Gd-flavone, **8b** Gd-chrysin, and **8c** Gd-galangin) were synthesized in the presence of GdCl_3_·6H_2_O and 1 M NaHCO_3_. Most of the reactions provided a good yield of 80% or more, and we established that the yields of Gd-galangin may differ (44.41% to 67.95%) depending on the reaction time, pH and temperature. The identities of synthesized materials were confirmed based on ^1^H NMR, ^13^C NMR, HR-FAB-MS, and melting point analyses. The purity of the Gd complexes (**8a**–**c**) were determined based on HPLC analysis (Figures S28, S30 and S32), and the presence of free gadolinium ions was determined using arsenazo III solution (Figure S33) [30]. The overall reaction scheme is shown in Figure 1.

### 3.2. Physicochemical Characterization

The longitudinal (*r*_1_) and transverse (*r*_2_) relaxivities of Gd-flavone, Gd-chrysin, Gd-galangin, Gd-BT-DO3A, and Gd-DOTA were estimated in water, PBS, and a 0.67 mM HSA solution (Table 1). Among the three synthesized compounds, Gd-galangin was found to be characterized by the greatest *r*_1_ relaxivity, whereas Gd-flavone, Gd-BT-DO3A, and Gd-DOTA were found to have similar values. Additionally, we found that Gd-chrysin in a solution with 0.67 mM HSA showed a slightly higher level of relaxation than the other compounds studied.

The octanol-water partition coefficient (*log P*) was used to calculate the relative polarities of Gd-flavone, Gd-chrysin, and Gd-galangin, for which we obtained values of −1.40, −0.91, and −0.74, respectively (Table 1). For comparison, we also determined the *log P* values of the clinically used agents Gd-BT-DO3A (−3.13) and Gd-DOTA (−3.09). Notably, we established that the lipophilicity of the three new Gd complexes was significantly higher than that of the gadolinium complexes currently used in clinical settings, which we speculate may be attributable to the presence of polyphenolic hydroxy groups. Given that *log P* values tend to be highly correlated with relaxivity and protein binding, it is conceivable that the high relaxivities of Gd-flavone, Gd-chrysin, and Gd-galangin may be associated with their high lipophilicities.

### 3.3. Kinetic Stability (Transmetalation Kinetics and pH Stability)

**Figure 2:**
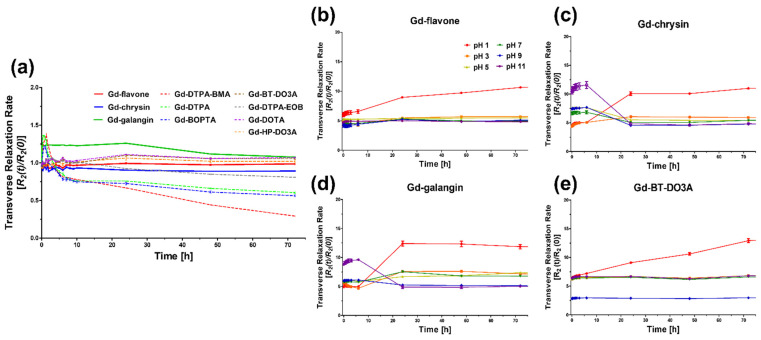
(**a**) Transmetalation kinetics stability of 1 mM Gd-flavone, Gd-chrysin, Gd-galangin and commercial MR contrast agents by transverse relaxation rate (*R*_2_) over time (*R*_2_(*t*)/*R*_2_(0)) as a function of time at 3.0 T for 72 h. (**b**–**e**) pH stability of Gd-flavone, Gd-chrysin, Gd-galangin and Gd-BT-DO3A. Samples prepared at the same concentration with various pH buffers were measured at 3.0 T. An additional pH stability test is shown as Figure S34.

Endogenous metal ions, including Zn^2+^, Cu^2+^, and Ca^2+^, can compete with Gd^3+^ ions for the relevant ligands, resulting in Gd ion loss, which can lead to diseases such as NSF or the deposition of Gd^3+^ ions in the brain [31]. Given that Zn^2+^ ions occur at higher blood concentrations (~100 μM) than other potential competitive ions, these are the most likely to compete with Gd^3+^ [32]. Compared with their linear type counterparts, such as Gd-DTPA and Gd-DTPA-EOB, Gd-based macrocyclic chelates are characterized by higher kinetic inertness. Among the evaluated complexes, we established that Gd-galangin had the highest kinetic inertness values, whereas Gd-flavone and Gd-chrysin, which have the same macrocyclic chelate structure as Gd-BT-DO3A and Gd-DOTA, were found to perform similarly. Whereas we detected no significant changes in the *R*_2_ relaxation rate of the complexes over 72 h of monitoring, we found that during the same period, the *R*_2_ values of the linear DTPA analogs underwent significant reductions (Figure 2a).

Furthermore, we assessed the *R*_2_ relaxivity-associated stability of Gd-flavone, Gd-chrysin, and Gd-galangin over time at different pH values ranging from 1 to 11 and Gd-BT-DO3A as a control (Figure S34). We accordingly established that the *R*_2_ values remained relatively constant across a broad spectrum of pH values ranging from 3 to 11, thereby indicating that the assessed Gd complexes are sufficiently stable under both extremely acidic and basic conditions. However, an increase in *R*_2_ values detected at pH 1 would tend to indicate that the enhanced relaxivity at an extremely low pH is attributable to complex dissociation. Comparable results were obtained for the commercial agent Gd-BT-DO3A. These findings nevertheless indicate that Gd-flavone, Gd-chrysin, and Gd-galangin retain sufficient stability for in vivo testing.

### 3.4. DPPH, FRAP, ABTS Free Radical Scavenging Activity

**Figure 3:**
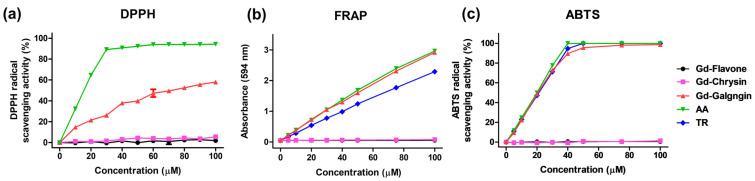
Radical scavenging activity of Gd-flavone, Gd-chrysin, Gd-galangin, ascorbic acid (AA) and trolox (TR). (**a**) 2,2-Diphenyl-1-picrylhydrazyl (DPPH), (**b**) Ferric-Reducing Antioxidant Potential (FRAP), (**c**) 2,2′-Azino-bis (3-ethylbenzthiazoline-6-sulphonic acid (ABTS).

As control groups for assessing the free radical-scavenging activity of the synthesized Gd complexes, we used the representative antioxidants ascorbic acid (AA) and Trolox (TR) (Figure 3). Neither Gd-flavone nor Gd-chrysin was found to remove radicals, whereas compared with AA, Gd-galangin at 30 μM showed a three-fold higher scavenging of DTTH, although the difference narrowed as the concentration increased. With respect to FRAP scavenging, this showed a better effect than TR and showed a similar tendency to AA. ABTS experiments showed patterns similar to AA and TR, and only Gd-galangin showed a radical removal effect comparable to the efficacies of AA and TR.

### 3.5. In Vitro Cell Toxicity

The toxicity of Gd-galangin was evaluated based on the use of a CCK cell viability assay (Figure S35). Whereas at concentrations up to 50 μM, the viability of RAW 264.7 macrophages was maintained at approximately 100%, at 75 μM, the viability had declined by approximately 60%.

### 3.6. In Vivo Elvaluation of MR Diagnosic Target in Inflammatoion

**Figure 4:**
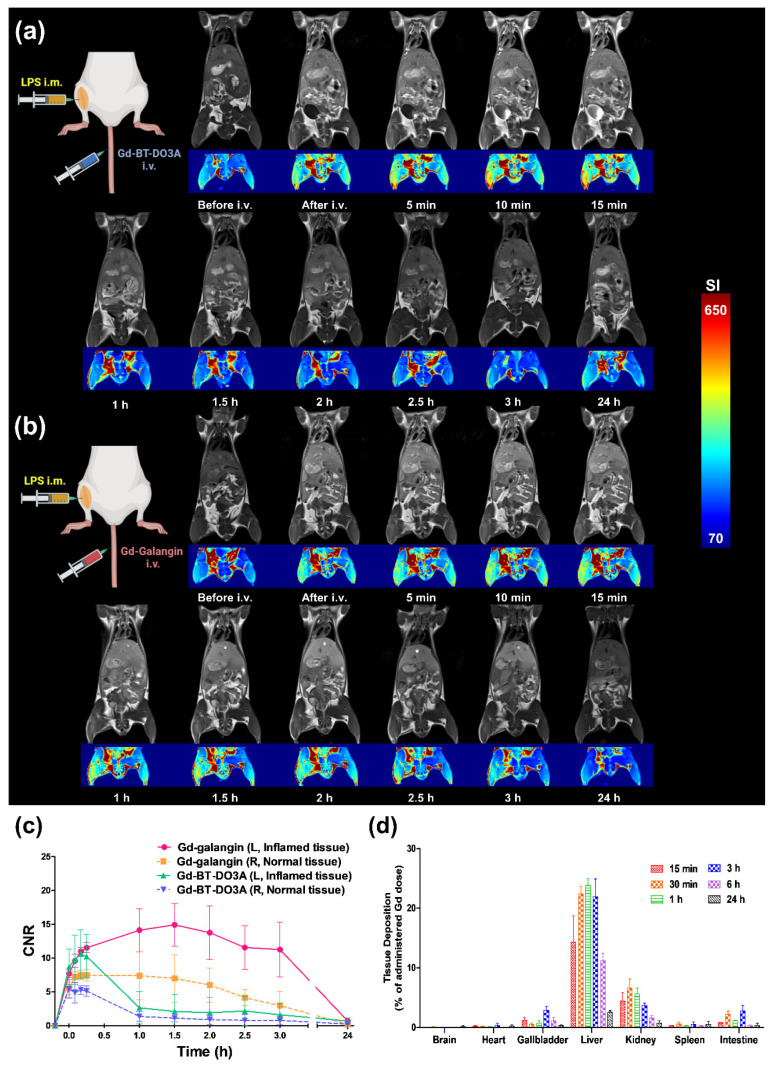
*T*_1_-weighted whole body magnetic resonance (MR) images of lipopolysaccharide (LPS)-induced inflammation animal model obtained after the intravenous injection of (**a**) Gd-BT-DO3A (Gadovist^®^) and (**b**) Gd-galangin, respectively (0.1 mmol Gd/kg, *n* = 3 for each group). (**c**) Contrast-to-ratio (CNR) of *T*_1_-weighted whole body MR images obtained after Gd-BT-DO3A (Gadovist^®^) and Gd-galangin intravenous injection (*n* = 3). Normal tissue (right leg) and inflamed tissue (left leg). CNR was calculated using Equation (3). (**d**) Biodistribution of Gd-galangin (0.1 mmol Gd/kg) in normal BALB/c mice by Gd percentage in each tissue. Groups of mice (*n* = 4) were sacrificed at 15 min, 30 min, 1 h, 3 h, 6 h and 24 h. Data are expressed as the mean ± SD.

The diagnostic imaging properties of Gd-galangin were evaluated using a mouse model of LPS-induced inflammation. Gd-galangin and Gd-BT-DO3A (as a control agent) were intravenously injected at the same concentration (0.1 mmol Gd/kg), and coronal and axial *T1*-weighted MR images of the whole body and inflammation sites were obtained at 3.0 T. MR imaging contrasts in the liver and inflammatory lesions revealed a significant difference between the Gd-galangin- and Gd-BT-DO3A-treated mice (Figure 4a,b). A 1 h following treatment, the signal intensity was low in the liver, although Gd-galangin maintained a high signal intensity for up to 3 h. This enhancement pattern was consistent with the biodistribution results (Figure 4d). Signal intensity in the gallbladder was enhanced 1 h after injection, and we observed that during the hepatobiliary phase, the contrast agent was absorbed by hepatocytes and then excreted via the biliary route, thereby indicating an enhancement pattern characteristic of a hepatobiliary excretion contrast agent. The flavonoid moiety of the Gd complex confers high lipophilicity, and Gd-galangin can maintain a higher and longer signal intensity in the liver and gallbladder. At the site of inflammation, the signal enhancement of Gd-galangin measured based on CNR continued to increase for up to 1.5 h, indicating a CNR difference that was approximately five-fold stronger than that obtained using Gd-BT-DO3A. In addition, signal enhancement was maintained for at least 3 h in the inflamed tissues (Figure 4c). Given the high lipophilicity of Gd-galangin, its retention time in the body is prolonged, thereby enhancing the targeting capacity of this agent for inflamed tissues, which is beneficial with respect to diagnosing inflammation using MRI.

### 3.7. In Vivo Evaluation of ROS Scavenging Affinity

**Figure 5:**
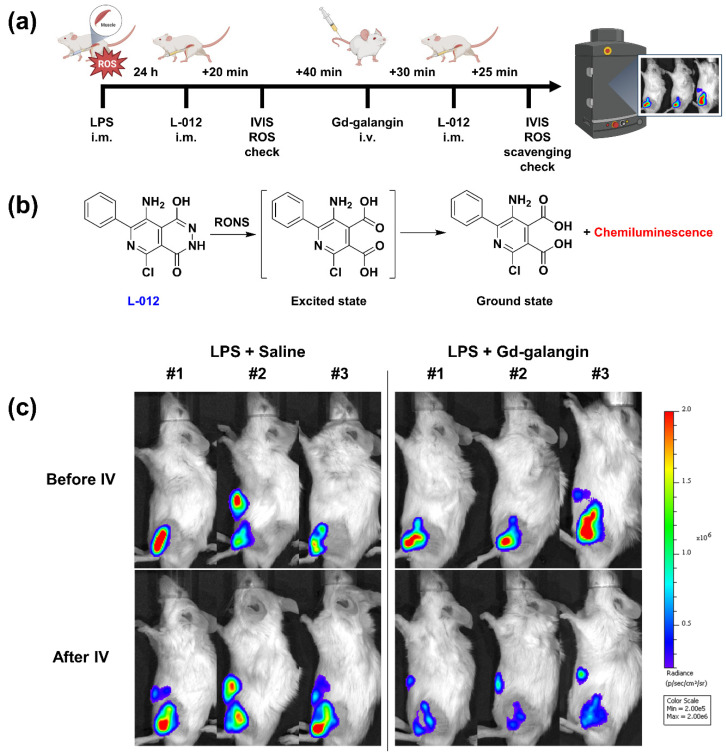
(**a**) Time points for intramuscular injection (i.m.) and intravenous injection (i.v.). (**b**) On the use of L-012, a luminol-based chemiluminescent probe, for detecting reactive oxygen (and nitrogen) species. (**c**) Chemiluminescence images of reactive oxygen species (ROS) using L-012.

The luminol derivative L-012 is a highly sensitive chemiluminescence material characterized by greater activity than luminol itself (Figure 5b) [33]. It reacts with different ROS generated in the body, although activity wanes after 40 min to 1 h within the body, and thus repeated injection in vivo is necessary [34]. This property can be used to detect ROS and monitor the ability of a given agent to remove ROS. In this experiment, we detected a luminescent signal at the site of intramuscular injection with LPS, thereby indicating that L-012 reacts with ROS generated in response to LPS-induced inflammation. Having established the production of ROS in our mice model, we assessed the scavenging properties of Gd-galangin. Compared with the intravenous injection of saline, which had no significant effect on ROS levels, a single administration of Gd-galangin promoted a marked reduction in levels, thereby revealing the potent capacity of Gd-galangin to eliminate inflammation-induced ROS in vivo (Figure 5c).

### 3.8. Effect of Gd-Galangin on LPS-Induced NO Production and iNOS Inhibition

**Figure 6:**
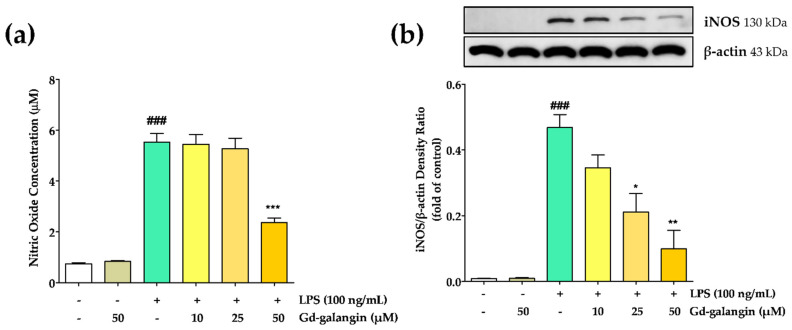
Effects of Gd-galangin on lipopolysaccharide (LPS)-stimulated (**a**) nitric oxide (NO) and (**b**) inducible nitric oxide synthase (iNOS) expression. (Data are shown as the mean ± SEM, *t*-test, *n* ≥ 3. ### *p* < 0.001 compared to control group; * *p* < 0.05, ** *p* < 0.01, *** *p* < 0.001 compared to LPS-stimulated group.).

Given that LPS stimulates iNOS expression in macrophages and Kupffer cells [35], we investigated the effect of Gd-galangin on RAW 264.7 macrophage cells (Figure 6). Compared with the untreated control cells, those treated with LPS alone showed a significantly elevated induction of NO production. Although we detected no inhibitory effect of Gd-galangin on NO production when administered at concentrations of 10 and 25 μM, significant reductions in NO levels were observed at 50 μM, and we established that this reduction was associated with the regulation of iNOS expression by Gd-galangin. In response to LPS treatment, we detected a significant increase in the expression of iNOS, whereas expression levels were reduced by Gd-galangin in a dose-dependent manner. These observations thus provide evidence to indicate that a Gd-galangin-mediated reduction in iNOS expression contributes to inhibiting LPS-induced NO production.

### 3.9. ROS Scavenging Effect of Gd-Galangin

**Figure 7:**
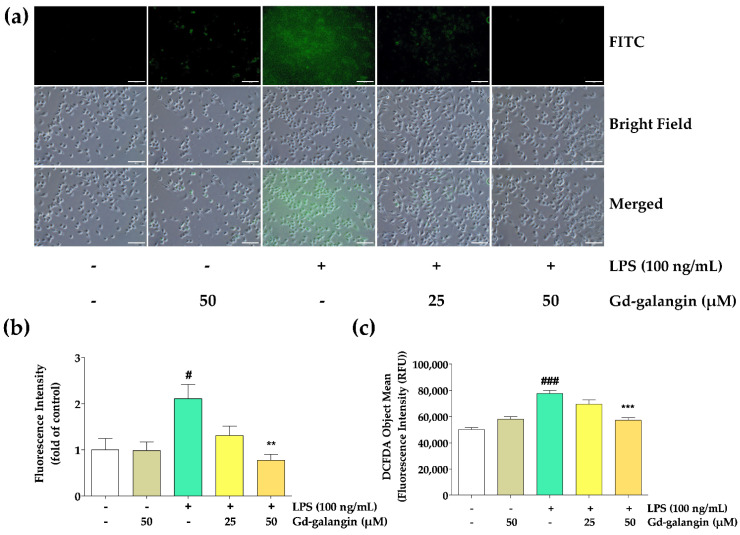
Reactive oxygen species (ROS)-scavenging effect of Gd-galangin on RAW 264.7 macrophage cells. Cellular ROS was determined by 2,7-dichlorofluoroscin diacetate (DCFDA) assay. (**a**) Images of RAW 264.7 cells captured with fluorescence microscope. Scale bar, 100 μm. (**b**) The fluorescence intensity of (**a**) cellular images. (**c**) The fluorescence intensity measured using a microplate reader. (Data are shown as the mean ± SEM, *t*-test, *n* ≥ 3. # *p* < 0.05, ### *p* < 0.001 compared to control group; ** *p* < 0.01, *** *p* < 0.001 compared to LPS-stimulated group.).

The ROS removal capacity of Gd-galangin was also assessed using a DCF-DA assay. After its diffusion through the cell membrane, DCF-DA is hydrolyzed by an esterase to generate the non-fluorescent DCF-H form. In the presence of cellular ROS, this product is rapidly oxidized to yield the fluorescent DCF form [36]. Whereas RAW 264.7 cells treated with LPS alone were characterized by high fluorescence intensity, we detected a dose-dependent reduction in intensity in the cells subjected to Gd-galangin treatments (Figure 7), thereby confirming the efficacy of Gd-galangin in eliminating the ROS generated in response to LPS-induced inflammation.

### 3.10. Anti-Inflammatory Effect of Gd-Galangin

**Figure 8:**
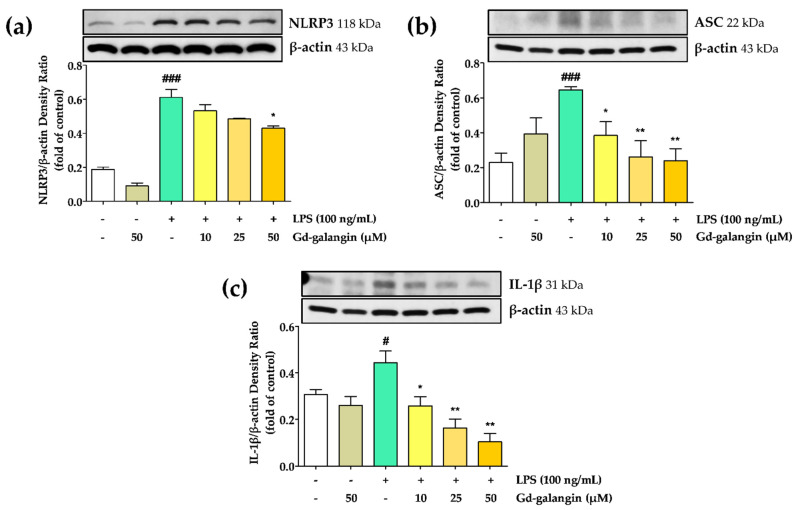
Anti-inflammatory effect of Gd-galangin in lipopolysaccharide (LPS)-induced RAW 264.7 cells. (**a**,**b**) Nucleotide binding and oligomerization domain-like receptor family pyrin domain-containing protein 3 (NLRP3)-related factors and (**c**) their product IL-1β were determined with Western blot analysis. (Data are shown as the mean ± SEM, *t*-test, *n* ≥ 3. # *p* < 0.05, ### *p* < 0.001 compared to control group; * *p* < 0.05, ** *p* < 0.01 compared to LPS-stimulated group).

To assess the inhibitory capacity of Gd-galangin with respect to NLRP3 inflammasome activation, we examined its ability to downregulate the expression of ASC, IL-1β, and NLRP3. On the treatment of RAW 264.7 cells with LPS alone, we detected increases in the expression of NLRP3 and ASC, which in turn can contribute to the promotion of NLRP3 inflammasome activation. The administration of Gd-galangin was observed to promote a slight downregulation of the NLRP3 expression induced by LPS (Figure 8a), whereas the elevated levels of ASC and IL-1β produced in response to LPS treatment were dose-dependently downregulated by Gd-galangin (Figure 8b,c). These findings thus indicate that Gd-galangin can inhibit the expression of NLRP3 and ASC and their pro-inflammatory product IL-1β, which by suppressing subsequent NLRP3 inflammasome activation that believed to have an anti-inflammatory effect.

### 3.11. In Vivo Anti-Inflammatory Effect of Gd-Galangin

**Figure 9:**
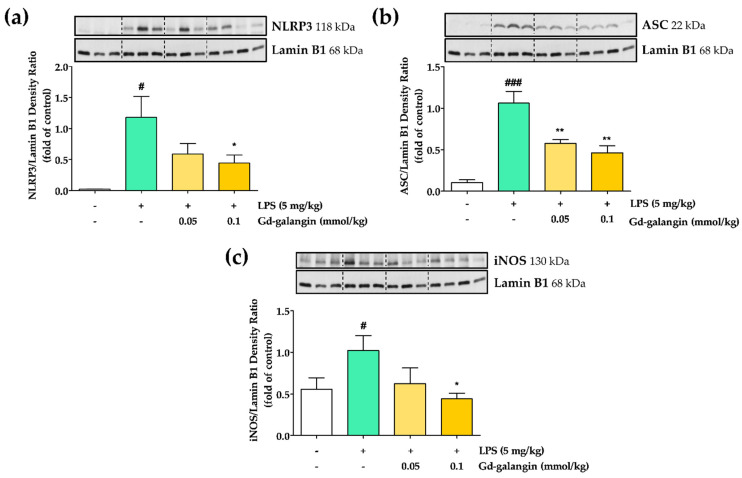
Anti-inflammatory effect in lipopolysaccharide (LPS)-induced thigh edema model. Thigh tissues were used to evaluate the expression of inflammatory factors with Western blot analysis. (**a**,**b**) shows about the expression of NLRP3-related factors. (**c**) shows about the expression of iNOS. (Data are shown as the mean ± SEM, *t*-test, *n* ≥ 3. # *p* < 0.05, ### *p* < 0.001 compared to control group; * *p* < 0.05, ** *p* < 0.01 compared to LPS-stimulated group).

The inhibitory effects of Gd-galangin on iNOS and NLPR3 inflammasome induction were also verified in an in vivo animal model. The mouse inflammation model developed in this study was based on the intramuscular injection of LPS into the thighs of BALB/c mice. At 6 h after LPS injection, Gd-galangin was intravenously administered at the same dose (0.1 Gd mmol/kg Gd-galangin) to facilitate MR imaging, and the inflamed tissue was excised 24 h later. Consistent with our in vitro observations using RAW 264.7 macrophage cells, we observed that LPS promoted the expression of the pro-inflammatory factor iNOS, which is associated with the production of nitric oxide (Figure 9a), and the NLRP3 inflammasome-related factors NLRP3 and ASC (Figure 9b,c), which were significantly downregulated in response to Gd-galangin. These findings accordingly indicate that Gd-galangin is also characterized by in vivo anti-inflammatory activity.

### 3.12. The Effect of Gd-Galangin on LPS-Induced MAPK Signaling Pathway

**Figure 10:**
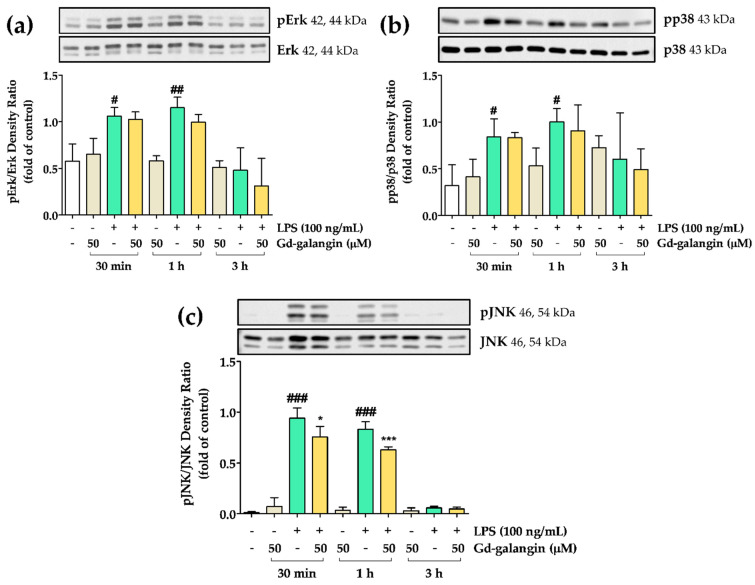
Effects of Gd-galangin on mitogen-activated protein kinase (MAPK) signaling pathway. (**a**,**b**) Gd-galangin had no effect on inhibiting phosphorylated Erk and p38. (**c**) Shows significant inhibition of phosphorylated p-c-Jun *N*-terminal kinase (JNK) by Gd-galangin. (Data are shown as the mean ± SEM, *t*-test, *n* ≥ 3. # *p* < 0.05, ## *p* < 0.01, ### *p* < 0.001 compared to control group; * *p* < 0.05, *** *p* < 0.001 compared to LPS-stimulated group.).

We also investigated the effects of Gd-galangin on the MAPK signaling pathway. In response to LPS stimulation of RAW 264.7 cells, we detected the phosphorylation of Erk, JNK, and p38, which was generally maintained at high levels for 30 min to 1 h. At 3 h post-treatment, however, all three of these MAPK pathway members showed low levels of phosphorylation. As shown in Figure 10a,b, although the administration of Gd-galangin did not significantly alter the levels of phosphorylated Erk and p38, it did effectively promote a reduction in the levels of phosphorylated JNK (Figure 10c). These findings thus tend to indicate that the anti-inflammatory activity of Gd-galangin is mediated, at least in part, via its inhibition of MAPK signaling, particularly with respect to JNK.

### 3.13. Anti-Inflammatory Effect of Gd-galangin through Inhibiting Translocation of NF-κB and Phosphorylation of IκBα

**Figure 11:**
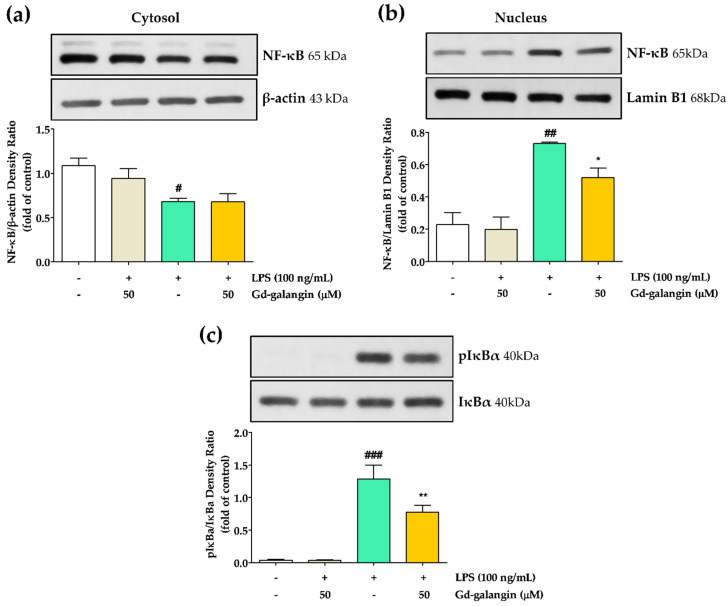
Effects of Gd-galangin on the nuclear transcription factor kappa B (NF-κB) signaling pathway. (**a**,**b**) Translocation of NF-κB has been confirmed with nuclear extraction before performing Western blot analysis. (**c**) Phosphorylation of nuclear factor of kappa light polypeptide gene enhancer in B-cells inhibitor (IκBα) has been developed at the cytosol level of RAW 264.7 cells. (Data are shown as the mean ± SEM, *t*-test, *n* ≥ 3. # *p* < 0.05, ## *p* < 0.01, ### *p* < 0.001 compared to control group; * *p* < 0.05, ** *p* < 0.01 compared to LPS-stimulated group).

In response to LPS treatment RAW 264.7 cells, we detected lower levels of cytoplasmic NF-κB compared with those in untreated cells, and a corresponding tendency to accumulate in the nucleus, thereby indicating that LPS stimulation promotes the nuclear translocation of NF-κB. Conversely, in response to Gd-galangin treatment, we established that the cytoplasmic levels of NF-κB were maintained, whereas there was a significant reduction in nuclear NF-κB (Figure 11a,b), thereby implying that Gd-galangin inhibits the nuclear translocation of NF-κB.

We also demonstrated that LPS promotes the phosphorylation of IκBα, whereas lower levels of phosphorylated IκBα were detected following the administration of Gd-galangin (Figure 11c). Collectively, these findings indicate that Gd-galangin inhibits the nuclear translocation of NF-κB via it repressive effect on the phosphorylation of IκBα.

### 3.14. Anti-Inflammatory Effect of Gd-Galangin through Nrf2 and HO-1 Expression

**Figure 12:**
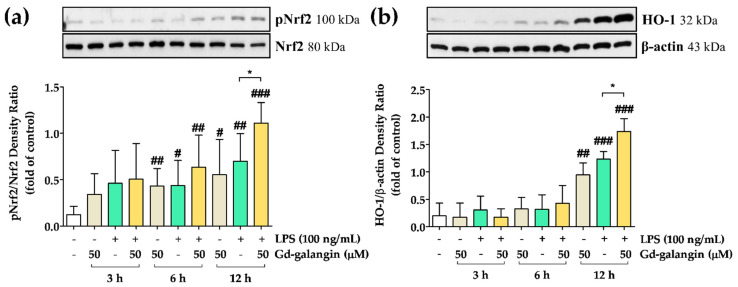
Nuclear factor erythroid-2-related factor 2 (Nrf2) and heme oxygenase-1 (HO-1) expression at the protein level in the RAW 264.7 cells. (**a**) Gd-galangin showed upregulation of phosphorylated Nrf2. (**b**) HO-1 was upregulated following phosphorylation of Nrf2 by Gd-galangin. The expression of Nrf2 and HO-1 was identified with Western blot analysis. Data are shown as the mean ± SEM, *t*-test, *n* ≥ 3. # *p* < 0.05, ## *p* < 0.01, ### *p* < 0.001 compared to control group; * *p* < 0.05 compared to LPS-stimulated group).

In response to oxidative stress, Nrf2 mediates HO-1 upregulation to activate the antioxidant reaction [37]. we proceeded to examine the involvement of the Nrf2 signaling pathway. The elevated phosphorylation of Nrf2 stimulated by LPS was further increased by treatment with Gd-galangin. At 12 h post-LPS stimulation, we detected a clear and statistically significant difference between the LPS-only treated group and the Gd-galangin-treated group (Figure 12a). With respect to HO-1 expression, whereas up to 6 h following LPS stimulation, there was no significant differences between the LPS-only- and Gd-galangin-treated groups, in response to the phosphorylation of Nrf2, we detected the upregulated expression of HO-1 at 12 h, with more pronounced increases being observed in the Gd-galangin-treated cells (Figure 12b). These results accordingly indicate that Gd-galangin has antioxidant effects by promoting the phosphorylation of Nrf2 and the upregulated expression of HO-1.

## 4. Discussion

This study was designed to evaluate the diagnostic ability of a contrast agent combined with flavonoids for inflammation to verify the antioxidant and anti-inflammatory effects. Numerous flavonoids and developed derivatives show that they are effective in preventing and treating inflammatory diseases such as encephalitis [38], hepatitis [39], and rheumatism [40], and serious diseases such as cancer [41], Alzheimer’s [42], and Parkinson’s disease [43]. The administration of the substances used in the study is common for oral administration, because most flavonoids have very low solubility or insoluble in water [44]. Oral administration is the most preferred method for drug administration due to convenience, patient compliance, and reduced risk of cross-infection [45]. However, oral drugs are mostly absorbed in the form of digestion or metabolism, and drugs with a maintained structure are absorbed into the body very limitedly [46]. It shows a bioavailability that is limited due to low permeability and stability, which severely reduces its effectiveness as a therapeutic agent [47]. In order to solve this problem, studies have been conducted to increase the solubility of drugs by combining polar groups or changing the structure [48]. However, the efficacy of the modified drug may be reduced or eliminated [49]. So, the new derivative of the drug must be designed by preserving the active site and verified through experiments. In several previous studies, DO3A-bound substances increased solubility in water, and high lipophilicity due to the combined moiety increased intracellular diffusion capacity. The improvement in solubility and lipophilicity of the synthesized DO3A-flavonoid allowed intravenous injection. This led to the expectation of fast and immediate anti-inflammatory effects of the flavonoids with a small amount of injection.

**Figure 13:**
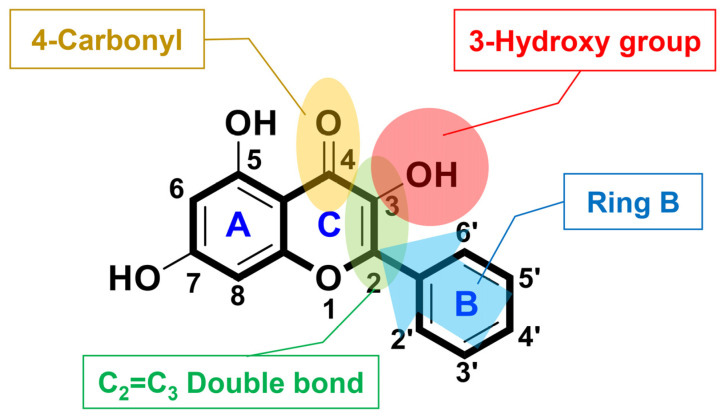
Structure of galangin and active site of antioxidant effect.

Among the basic frameworks of flavonoids (Figure 13), the 4-carbononyl group, C_2_=C_3_ double bond, and the hydroxyl group at position C_3_ are very important for radical removal and antioxidant function through synergistic effects [50,51]. Ring B is attributed to the π-π conjugation with chromone moiety, providing more resonance and coupling sites, and supporting chromone [52]. The linking group synthesis used the specific high reactivity of the hydroxyl group at position seven, and the ester bonding with DO3A through the linking group allowed for material synthesis in a good yield. DO3A-flavonoid is designed to maintain active sites for antioxidant effects and increase synthesis efficiency (Figure 1). The ester and ether bonds have a lower binding strength compared to the widely used amide bonds [16,34], and ester bonds can be decomposed into enzymes such as esterase [53]. Based on the ester bond, the DO3A and flavonoid portions are protected in decomposition because they have a large structure that is difficult to act on esterase [54], and the stability of DO3A-flavonoid was verified in experiments compared with used contrast agents (Figure 2). In recent studies, linker strategy experiments comparing amide bonds and ester bonds have shown contributions to permeability and cell activity [55]. These results show the advantage of using ester bonds. Flavone, chrysin, and galangin have differences in the position and number of hydroxyl groups for rings A and C. Considering the aforementioned structural features, the greatest effect could be expected in galangin, which was also confirmed in the radical-scavenging test (DPPH, FRAP, ABTS) executed with newly synthesized structures (Figure 3). The results of Gd-flavone and Gd-chrysin show the importance of the hydroxyl group at position three.

**Figure 14:**
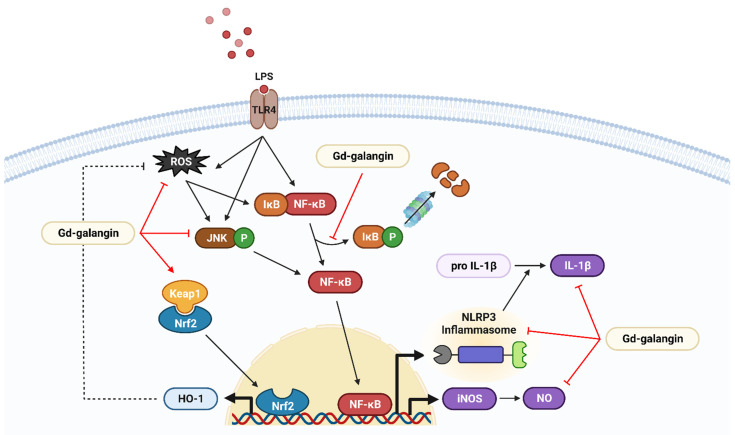
Anti-inflammatory signal pathway of Gd-galangin.

Based on this, we focused on Gd-galangin, Antioxidant and anti-inflammatory effects on LPS-induced inflammation were confirmed in in vitro and in vivo. The proposed mechanisms underpinning the effectiveness of Gd-galangin are summarized in Figure 14.

The inflammatory response is an important response for survival dominated by inflammatory cytokines and chemokines. In previous studies, galangin regulated the sub-factors NO and IL-1β while inhibiting iNOS and NLRP3 inflammasome expression (Figure 6 and Figure 8). In particular, NLRP3 inflammasome activation is targeted for inflammatory treatment because it promotes inflammation and induces disease development [56,57]. IL-1β is an inflammatory cytokine that matures by activated NLRP3 inflammation [58]. Gd-galangin reduced IL-1β expression due to its contribution to NLRP3 and ASC inhibition (Figure 9). Under normal conditions, IκBα binds to NF-κB, thereby suppressing its translocation to the nucleus. Contrastingly, under conditions of LPS-induced stress, phosphorylation of IκBα triggers the release of NF-κB, thereby restoring nuclear translocation [59]. In light of the previously reported performance of galangin, Gd-galangin also shows that inhibition of phosphorylation of IκBα inhibits NF-κB potential to the nucleus (Figure 11). In response to oxidative stress, Nrf2 mediates the upregulation of HO-1, thereby initiating antioxidant activity. This upregulated expression of HO-1 contributes to the maintenance of redox homeostasis via multiple mechanisms [37]. In these processes, Keap1 generally binds to Nrf2, thereby suppressing its translocation to the nucleus. Given that it has been established that phosphorylation of Nrf2 promotes its dissociation from Keap1, this thereby contributes to the expression of HO-1 [60]. According to previously reported papers, Gd-galangin exerts antioxidant effects by promoting the phosphorylation of Nrf2 and upregulating HO-1 expression (Figure 12). The MAPK signaling pathway is a cascade of serine/threonine kinases that regulate cell survival and death [61]. Erk, JNK, and p38 are the members of MAPK family which contribute to LPS-stimulated inflammation by phosphorylation [62,63]. Galangin showed a tendency to inhibit Erk, JNK, and p38, but Gd-galangin showed particularly prominent anti-inflammatory activity against JNK (Figure 10). There is a limitation to directly compare the anti-inflammatory effects of galangin and Gd-galangin. However, in this study, Gd-galangin sufficiently proved its antioxidant and anti-inflammatory effects. Considering that inflammation is an important hospital factor for various diseases, the possibility of treatment for various inflammatory diseases can be supported.

In this study, Gd-galangin, developed as a *T*_1_ MR contrast agent, had up to five times better enhancement than commercial contrast agents for inflammatory tissue, and maintains durability for more than 3 h. These features were caused by the combination with galangin, which supports the diagnostic ability for inflammation.

## 5. Conclusions

In this study, we synthesized and evaluated the properties of inflammation-targeted gadolinium compounds conjugated with flavonoids as potential theranostic agents for the treatment of LPS-induced inflammation. Among the assessed agents, Gd-galangin was found to show inflammation-specific MR contrast enhancement, thereby demonstrating the efficacy of this complex as an inflammation-targeting molecular imaging agent. We also established that Gd-galangin has strong anti-inflammatory effects, by inhibiting several pro-inflammatory mediators, including the NLRP3 inflammasome. Furthermore, our characterization of Gd-galangin indicates that complexes comprising flavonoids linked to Gd-based imaging agents have promising antioxidant efficacy. Based on these findings, we believe that flavonoid-conjugated gadolinium compounds, which combine imaging agents and flavonoids as inflammation-targeting and anti-inflammatory drugs, can be applied as an extremely efficient single theranostic platform for the treatment of inflammation.

## Figures and Tables

**Table 1:** Relaxivity and octanol-water partition coefficients of Gd-flavone, Gd-chrysin, Gd-galangin, Gd-BT-DO3A (Gadovist^®^), Gd-HP-DO3A (ProHance^®^), Gd-DTPA-BMA (Omniscan^®^), Gd-DOTA (Dotarem^®^), Gd-DTPA (Magnevist^®^), Gd-BOPTA (Multihance^®^) and Gd-DTPA-EOB (Primovist^®^) in water, PBS and HSA at 3.0 T, 293 K.

Contrast Agents	*r*_1_ (mM^−1^ s^−1^)			*r*_2_ (mM^−1^ s^−1^)			log *P* _oct/wat_
	Water	PBS	HSA	Water	PBS	HSA	
Gd-flavone	3.70 ± 0.21	3.94 ± 0.06	4.11 ± 0.08	4.35 ± 0.21	4.30 ± 0.10	7.29 ± 0.08	−1.40
Gd-chrysin	4.16 ± 0.22	4.41 ± 0.07	4.82 ± 0.10	4.82 ± 0.27	5.41 ± 0.13	10.11 ± 0.11	−0.91
Gd-galangin	4.76 ± 0.30	4.13 ± 0.09	4.53 ± 0.07	5.85 ± 0.44	6.83 ± 0.32	12.19 ± 0.19	−0.74
Gd-BT-DO3A	3.80 ± 0.16	4.57 ± 0.06	4.30 ± 0.08	4.62 ± 0.15	5.20 ± 0.05	6.51 ± 0.07	−3.13
Gd-DOTA	3.33 ± 0.14	3.99 ± 0.04	-	3.87 ± 0.14	4.31 ± 0.15	-	−3.09
Gd-BOPTA	4.75 ± 0.17	5.49 ± 0.07	-	5.33 ± 0.20	6.13 ± 0.13	-	−2.90
Gd-DTPA-EOB	6.07 ± 0.22	7.46 ± 0.08	-	6.86 ± 0.20	8.27 ± 0.05	-	−3.19
Gd-HP-DO3A	3.29 ± 0.15	4.04 ± 0.08	-	4.24 ± 0.13	4.44 ± 0.17	-	-
Gd-DTPA-BMA	3.33 ± 0.14	3.98 ± 0.06	-	4.00 ± 0.30	4.72 ± 0.08	-	-
Gd-DTPA	3.77 ± 0.20	4.59 ± 0.07	-	4.55 ± 0.29	5.50 ± 0.07	-	-

Phosphate-buffered saline (PBS) (pH 7.4) and human serum albumin (HSA) (0.67 mM) were used. Values are expressed as mean ± SD (*n* = 3).

## Data Availability

Not applicable.

## Supplementary Materials

The following supporting information can be downloaded at: https://www.mdpi.com/article/10.3390/antiox11122470/s1, Figure S1: ^1^H NMR spectrum of compound **2**; Figure S2: ^13^C NMR spectrum of compound **2**; Figure S3: High resolution FAB-mass spectrum of compound **2**; Figure S4: ^1^H NMR spectrum of compound **3**; Figure S5: ^13^C NMR spectrum of compound **3**; Figure S6: High resolution FAB-mass spectrum of compound **3**; Figure S7: ^1^H NMR spectrum of compound **1a**; Figure S8: ^13^C NMR spectrum of compound **1a**; Figure S9: High resolution FAB-mass spectrum of compound **1a**; Figure S10: ^1^H NMR spectrum of compound **1b**; Figure S11: ^13^C NMR spectrum of compound **1b**; Figure S12: High resolution FAB-mass spectrum of compound **1b**; Figure S13: ^1^H NMR spectrum of compound **1c**; Figure S14: ^13^C NMR spectrum of compound **1c**; Figure S15: High resolution FAB-mass spectrum of compound **1c**; Figure S16: ^1^H NMR spectrum of compound **5**; Figure S17: ^13^C NMR spectrum of compound **5**; Figure S18: ^1^H NMR spectrum of compound **7a**; Figure S19: ^13^C NMR spectrum of compound **7a**; Figure S20: High resolution FAB-mass spectrum of compound **7a**; Figure S21: ^1^H NMR spectrum of compound **7b**; Figure S22: ^13^C NMR spectrum of compound **7b**; Figure S23: High resolution FAB-mass spectrum of compound **7b**; Figure S24: ^1^H NMR spectrum of compound **7c**; Figure S25: ^13^C NMR spectrum of compound **7c**; Figure S26: High resolution FAB-mass spectrum of compound **7c**; Figure S27: High resolution FAB-mass spectrum of compound **8a**; Figure S28: HPLC spectrum of compound **8a**; Figure S29: High resolution FAB-mass spectrum of compound **8b**; Figure S30: HPLC spectrum of compound **8b**; Figure S31: High resolution FAB-mass spectrum of compound **8c**; Figure S32: HPLC spectrum of compound **8c**; Figure S33: Measuring of free gadolinium by arsenazo III; Figure S34: pH Stability of Gd-flavone, Gd-chrysin, Gd-galangin and commercial MR contrast agents; Figure S35: Cell viability of RAW 264.7 mouse macrophage cell in various concentration of Gd-galangin; Figure S36: Full band for all western blot experiments [64,65,66,67].

## Abbreviations

ROS	reactive oxygen species
iNOS	inducible nitric oxide synthase
NF-κB	nuclear transcription factor kappa B
TNF-α	tumor necrosis factor-α
IL-1β	interleukin-1β
IL-6	interleukin-6
NO	nitric oxide
JNK	p-c-Jun *N*-terminal kinase
IκBα	nuclear factor of kappa light polypeptide gene enhancer in B-cells inhibitor
Erk	extracellular signal-regulated kinase
HO-1	heme oxygenase-1
Nrf2	nuclear factor erythroid-2-related factor 2
NLRP3	nucleotide binding and oligomerization domain-like receptor family pyrin domain-containing protein 3
MAPK	mitogen-activated protein kinase
LPS	lipopolysaccharide
MRI	magnetic resonance imaging
HSA	human serum albumin
SE	spin echo
TE	echo time
NEX	number of excitations
FOV	field of view
TR	repetition time
SNR	signal-to-noise ratio
CNR	contrast-to-noise ratio
NSF	nephrogenic systemic fibrosis
