# Supplementary material for: Flavonoid-Conjugated Gadolinium Complexes as Anti-Inflammatory Theranostic Agents
Source: Antioxidants (Basel). 2022 Dec 15;11(12):2470. doi: 10.3390/antiox11122470 (PMC9774776; doi:10.3390/antiox11122470)
Supplement: Supplementary file 1 [file antioxidants-11-02470-s001.zip › antioxidants-2060379-supplementary.pdf]

Supplementary Materials

Flavonoid−Conjugated Gadolinium Complexes as Anti−Inflammatory Theranostic Agents

Byeong Woo Yang [1], Sohyeon Yang [2], Soyeon Kim [3], Ah Rum Baek [4], Bokyung Sung [1], Yeoun-Hee Kim [5], Jung Tae Lee [4], Sang Yun Lee [1], Hee-Kyung Kim [6], Garam Choi [5], Ji-Ae Park [3], Sung-Wook Nam [7], Gang-Ho Lee [8] and Yongmin Chang [1,2,4,7,9,*]

[1] Department of Medical & Biological Engineering, Kyungpook National University, 80 Daehak−ro, Buk−gu, Daegu 41566, Republic of Korea
[2] Department of Medical Science, School of Medicine, Kyungpook National University, 680 Gukchaebosang−ro, Jung−gu, Daegu 41944, Republic of Korea
[3] Division of Applied RI, Korea Institute of Radiological & Medical Sciences, (KIRAMS), 75 Nowon−ro, Nowon−gu, Seoul 01812, Republic of Korea
[4] Institute of Biomedical Engineering Research, Kyungpook National University, 680 Gukchaebosang−ro, Jung−gu, Daegu 41944, Republic of Korea
[5] R&D Center, Etnova Therapeutics Corp., 124, Sagimakgol−ro, Jungwon−gu, Seongnam−si 13207, Republic of Korea
[6] Preclinical Research Center, Daegu−Gyeongbuk Medical Innovation Foundation, 88 Dongnae−ro, Dong−gu, Daegu 41061, Republic of Korea
[7] Department of Molecular Medicine, School of Medicine, Kyungpook National University, 680 Gukchaebosang−ro, Jung−gu, Daegu 41944, Republic of Korea
[8] Department of Chemistry, Kyungpook National University, 80, Daehak−ro, Buk−gu, Daegu 41566, Republic of Korea
[9] Department of Radiology, Kyungpook National University Hospital, 130 Dongdeok−ro, Jung−gu, Daegu 41944, Republic of Korea
* Correspondence: ychang@knu.ac.kr; Tel.: +82−53−420−5471

Contents

1. **Synthesis and Characterization**

Figure S1. ^{1}H NMR spectrum of compound **2**

Figure S2. ^{13}C NMR spectrum of compound **2**

Figure S3. High resolution FAB-mass spectrum of compound **2**

Figure S4. ^{1}H NMR spectrum of compound **3**

Figure S5. ^{13}C NMR spectrum of compound **3**

Figure S6. High resolution FAB-mass spectrum of compound **3**

Figure S7. ^{1}H NMR spectrum of compound **1a**

Figure S8. ^{13}C NMR spectrum of compound **1a**

Figure S9. High resolution FAB-mass spectrum of compound **1a**

Figure S10. ^{1}H NMR spectrum of compound **1b**

Figure S11. ^{13}C NMR spectrum of compound **1b**

Figure S12. High resolution FAB-mass spectrum of compound **1b**

Figure S13. ^{1}H NMR spectrum of compound **1c**

Figure S14. ^{13}C NMR spectrum of compound **1c**

Figure S15. High resolution FAB-mass spectrum of compound **1c**

Figure S16. ^{1}H NMR spectrum of compound **5**

Figure S17. ^{13}C NMR spectrum of compound **5**

Figure S18. ^{1}H NMR spectrum of compound **7a**

Figure S19. ^{13}C NMR spectrum of compound **7a**

Figure S20. High resolution FAB-mass spectrum of compound **7a**

Figure S21. ^{1}H NMR spectrum of compound **7b**

Figure S22. ^{13}C NMR spectrum of compound **7b**

Figure S23. High resolution FAB-mass spectrum of compound **7b**

Figure S24. ^{1}H NMR spectrum of compound **7c**

Figure S25. ^{13}C NMR spectrum of compound **7c**

Figure S26. High resolution FAB-mass spectrum of compound **7c**

Figure S27. High resolution FAB-mass spectrum of compound **8a**

Figure S28. HPLC spectrum of compound **8a**

Figure S29. High resolution FAB-mass spectrum of compound **8b**

Figure S30. HPLC spectrum of compound **8b**

Figure S31. High resolution FAB-mass spectrum of compound **8c**

Figure S32. HPLC spectrum of compound **8c**

Figure S33. Absorbance spectra for free gadolinium verification using arsenazo III

2. Radical Scavenging Study

2.1. 2,2-Diphenyl-1-picrylhydrazyl (DPPH) Radical Scavenging Activity

2.2. Ferric Reducing Antioxidant Potential (FRAP) Radical Scavenging Activity

2.3. 2,2′-Azino-bis (3-ethylbenzthiazoline-6-sulphonic acid (ABTS) Radical Scavenging Activity

3. Other Figures

Figure S34. pH Stability of Gd-flavone, Gd-chrysin, Gd-galangin and commercial MR contrast agents.

Figure S35. Cell viability of RAW 264.7 mouse macrophage cell in various concentration of Gd-galangin.

Figure S36. Full band for all western blot experiments.

1. Synthesis and Characterization

4-Oxo-2-phenyl-4H-chromene-3,5,7-triyl triacetate (2).

2 prepared as described previously [30,31]. Galangin (3,5,7-trihydroxyflavone) (5.4328 g, 20 mmol) was dissolved in THF (200 mL) and triethylamine (34 mL, 240 mmol). The mixture was added acetic anhydride (24 mL, 240 mmol) and stirred at room temperature for 24-48 h. The reaction was monitored by TLC (hexane : ethyl acetate = 1 : 1) and stirred until the disappearance of the starting material. Removal of the solvent left a crude mixture, which was diluted with ethyl acetate (300 mL) and water (100 mL). The Aqueous layer was controlled to pH 7 with 5% $NaHCO_3$ solution, extracted with ethyl acetate, dried Na_2SO_4 and concentrated. The residue was washed with 10% ethyl acetate/hexane (v/v). The product (2) was obtained as a white solid (7.160 g, 90%). mp 146-148 °C. ^{1}H NMR (500 MHz, $CDCl_3$): δ (ppm) 7.79-7.81 (m, 2H), 7.48-7.54 (m, 3H), 7.33 (d, *J* = 2.2 Hz, 1H), 6.87 (d, *J* = 2.2 Hz, 1H), 2.44 (s, 3H), 2.33 (s, 3H), 2.31 (s, 3H); ^{13}C NMR (125 MHz, $CDCl_3$): δ (ppm) 170.37, 169.44, 168.04, 168.02, 157.16, 155.79, 154.33, 150.56, 134.02, 131.60, 129.65, 128.91, 128.32, 114.98, 113.93, 109.20, 21.32, 21.22, 20.72. HR-FAB-MS (EI) Anal. Calc. for $C_{21}H_{18}O_8$ (MH^+): 397.0923. Found: 397.0925.

7-Hydroxy-4-oxo-2-phenyl-4H-chromene-3,5-diyl diacetate (3).

3 prepared as described previously [30,31]. **2** (7.927 g, 20 mmol) was dissolved in N-methyl-2-Pyrrolidone (NMP) (150 mL) under N_2 and the solution was cooled to 0 °C. After adding thiophenol (2.644 g, 24 mmol) while maintaining temperature, imidazole (0.477 g, 7 mmol) dissolved in NMP was syringed. The mixture was slowly raised to room temperature and reacted for 6-12 h. The reaction was monitored by TLC (hexane : ethyl acetate = 1 : 1) and stirred until the disappearance of the starting material. The mixture was diluted with ethyl acetate, and the washed with 1 M HCl and brine. The organic layer was dried over Na_2SO_4 and concentrated. The residue was washed with 10% ethyl acetate/hexane and 10% IPA/hexane (v/v). The product (3) was obtained as white or light yellow solid (13.122 g, 93%). mp 222-224 °C. ^{1}H NMR (500 MHz, DMSO-d_6): δ (ppm) 11.29 (s, 1H), 7.83-7.86 (m, 2H), 7.57-7.60 (m, 3H), 6.93 (d, *J* = 2.3 Hz, 1H), 6.65 (d, *J* = 2.3 Hz, 1H), 2.31 (s, 3H), 2.28 (s, 3H); ^{13}C NMR (125 MHz, DMSO-d_6): δ (ppm) 169.46, 169.17, 168.34, 163.15, 158.04, 154.39, 150.64, 133.04, 131.81, 129.57, 129.37, 128.27, 109.63, 109.48, 101.34, 21.27, 20.66. HR-FAB-MS (EI) Anal. Calc. for $C_{19}H_{16}O_7$ (MH^+): 355.0818. Found: 355.0820.

General procedure for the synthesis of 1.

1 prepared as described previously. To solution of 1,3-dibromopropane (10 eq) and K_2CO_3 (3 eq) in acetone, a solution of flavonoid (1 eq) in acetone was added dropwise. The resulting mixture was stirred for 4 h at 60 °C under N_2. The mixture was then cooled to room temperature, and filtered through a short column of silica gel (5% methanol/CH_2Cl_2, v/v) to remove impurities including inorganic salts. The solvent was removed, and the mixture wash slowly precipitated at low temperature under CH_2Cl_2/hexane condition to isolate 1,3-dibromopropane. Purified using column chromatography on silica gel (0-5% methanol/CH_2Cl_2, v/v) to give **1a-c**.

7-(3-Bromopropoxy)-2-phenyl-4H-chromen-4-one (1a).

To solution of 1,3-dibromopropane (5 mL, 50 mmol) and K_2CO_3 (2.073 g, 15 mmol) in acetone (50 mL), a solution of 7-hydroxyflavone (1.191 g, 5 mmol) in acetone (50 mL) was added dropwise. The resulting mixture was stirred for 4 h at 60 °C under N_2. Light yellow solid (1.286 g, 71.60%). mp 126-128 °C. ^{1}H NMR (500 MHz, $CDCl_3$): δ (ppm) 8.11 (d, *J* = 9.4 Hz, 1H), 7.87-7.88 (m, 2H), 7.49-7.50 (m 3H), 6.95-6.96 (m, 2H), 6.73 (s, 1H), 4.21 (t, *J* = 5.8 Hz, 2H), 3.62 (t, J = 6.3 Hz, 2H), 2.37 (qui, *J* = 6.0 Hz, 2H); ^{13}C NMR (125 MHz, $CDCl_3$): δ (ppm) 177.90, 163.34, 163.15, 158.06, 131.95, 131.58, 129.16, 127.24, 126.29, 118.15, 114.79, 107.67, 101.19, 66.13, 32.13, 29.75. HR-FAB-MS (EI) Anal. Calc. for $C_{18}H_{17}BrO_3$ (MH^+): 359.0283. Found: 359.0286.

7-(3-Bromopropoxy)-5-hydroxy-2-phenyl-4H-chromen-4-one (1b).

To solution of 1,3-dibromopropane (5 mL, 50 mmol) and K_2CO_3 (2.073 g, 15 mmol) in acetone (50 mL), a solution of chrysin (5,7-dihydroxyflavone) (1.271 g, 5 mmol) in acetone (50 mL) was added dropwise. The resulting mixture was stirred for 4 h at 60 °C under N_2. Light yellow solid (1.581 g, 84.25%). mp 156-157 °C. ^{1}H NMR (500 MHz, $CDCl_3$): δ (ppm) 12.69 (s, 1H), 7.84-7.86 (m, 2H), 7.48-7.55 (m, 3H), 6.62 (s, 1H), 6.48 (d, *J* = 2.2 Hz, 1H), 6.33 (d, *J* = 2.2 Hz, 1H), 4.17 (t, *J* = 5.8 Hz, 2H), 3.60 (t, *J* = 6.4 Hz, 2H), 2.34 (qui, *J* = 6.1 Hz, 2H); ^{13}C NMR (125 MHz, $CDCl_3$): δ (ppm) 182.56, 164.75, 164.09, 162.31, 157.88, 132.00, 131.38, 129.23, 126.40, 105.98, 98.80, 93.18, 66.11, 32.10, 29.74. HR-FAB-MS (EI) Anal. Calc. for $C_{18}H_{16}BrO_4$ (MH^+): 375.0232. Found: 375.0229.

7-(3-Bromopropoxy)-4-oxo-2-phenyl-4H-chromene-3,5-diyl diacetate (1c).

To solution of 1,3-dibromopropane (10 mL, 100 mmol) and K_2CO_3 (4.146 g, 30 mmol) in acetone (100 mL), a solution of **2** (3.543 g, 10 mmol) in acetone (100 mL) was added dropwise. The resulting mixture was stirred for 4 h at 60 °C under N_2. Light yellow solid (3.756 g, 79.02%). mp 122-124 °C. ^{1}H NMR (500 MHz, $CDCl_3$): δ (ppm) 7.79-7.81 (m, 2H), 7.47-7.53 (m, 3H), 6.87 (d, *J* = 2.4 Hz, 1H), 6.64 (d, *J* = 2.4 Hz, 1H), 4.20 (t, *J* = 5.8 Hz, 2H), 3.59 (t, *J* = 6.3 Hz, 2H), 2.43 (s, 3H), 2.36 (qui, *J* = 6.1 Hz, 2H), 2.30 (s, 3H). ^{13}C NMR (125 MHz, $CDCl_3$): δ (ppm) 170.33, 169.72,

168.20, 162.91, 158.37, 155.17, 150.91, 133.83, 131.37, 129.92, 128.86, 128.26, 111.43, 109.09, 99.58, 66.45, 31.97, 29.51, 21.30, 20.77. HR-FAB-MS (EI) Anal. Calc. for $C_{22}H_{20}BrO_7$ (MH^+): 475.0392. Found: 475.0391.

Tri-tert-butyl 2,2',2''-(10-(2-ethoxy-2-oxoethyl)-1,4,7,10-tetraazacyclododecane-1,4,7-triyl)triacetate (4) and **2-(4,7,10-tris(2-(tert-butoxy)-2-oxoethyl)-1,4,7,10-tetraazacyclododecan-1-yl)acetic acid (5).**

4, 5 prepared as described previously [66]. Tri-tert-butyl 2,2',2''-(1,4,7,10-tetraazacyclododecane-1,4,7-triyl)triacetate (10.0 g, 19 mmol) and K_2CO_3 (5.4 g, 38 mmol) were dissolved in acetonitrile (200 mL). Then ethyl bromoacetate (3.2 g, 19 mmol) in acetonitrile (20 mL) was added. The resulting mixture was stirred for 12 h at 70 °C under N_2. The mixture was then cooled to room temperature, and filtered through a short column of silica gel (acetonitrile) to remove impurities including inorganic salts. After the solvent was removed, proceed to the next step. **4** Crude product was dissolved in the mixture (400 mL) of 1,4-dioxane and 0.4 M NaOH with the ratio of 3:1 (v:v). This mixture was stirred for about 4 h under N_2 at 50 °C. After the solvent was removed, this mixture was dissolved in water (100 mL), and 1 M HCl was added slowly so that the solution was neutral. The mixture was extracted with CH_2Cl_2 (3 x 200 mL). The organic phases washed with brine (2 x 100 mL). After the solvent was removed, this product was recrystallized from diethyl ether. ^{1}H NMR (500 MHz, $CDCl_3$): δ (ppm) 2.82-3.52 (very broad peak, 8H), 1.78-2.77 (very broad peak, 16H), 1.40-1.41 (m, 27H). ^{13}C NMR (125 MHz, $CDCl_3$): δ (ppm) 175.19, 172.01, 171.75, 81.85, 81.77, 59.48, 56.39, 55.90, 50.81 (broad peak), 28.23, 28.13.

General procedure for the synthesis of 6-7.

5 (1 eq) was dissolved in THF and triethylamine (3 eq) was added. The solution of **1** (1.2 eq) in THF was slowly added and stirred at 40 °C for 2 h. The mixture was then cooled to room temperature, and filtered through a short column of silica gel (5% methanol/CH_2Cl_2, v/v) to remove inorganic impurities. Purified using column chromatography on silica gel (0-5% methanol/CH_2Cl_2, v/v) to give **6a-c**. **6** was dissolved in 1,4-dioxane and HCl was slowly added at 0 °C. The reaction was stirred and slowly allowed to reach 40 °C. After 12 h, removal of the solvent left a crude mixture, which was diluted with water and filtered. Purified using flash column chromatography on C18 silica (5-95% acetonitrile/water) to give as **7a-c**.

Tri-tert-butyl 2,2',2''-(10-(2-oxo-2-(3-((4-oxo-2-phenyl-4H-chromen-7-yl)oxy)propoxy)ethyl)-1,4,7,10-tetraazacyclododecane-1,4,7-triyl)triacetate (6a). 2,2',2''-(10-(2-Oxo-2-(3-((4-oxo-2-phenyl-4H-chromen-7-yl)oxy)propoxy)ethyl)-1,4,7,10-tetraazacyclododecane-1,4,7-triyl)triacetic acid (7a).

5 (3.226 g, 5.633 mmol) was dissolved in THF (50 mL) and triethylamine (1.601 g, 15.810 mmol) was added. The solution of **1a** (2.441 g, 6.792 mmol) in THF (50 mL) was slowly added and stirred at 40 °C for 2 h. **6a** was dissolved in 1,3-dioxane (20 mL), and HCl (10 mL) was slowly added at 0 °C. The reaction was stirred and slowly allowed to reach 40 °C. After 12 h, removal of the solvent left a crude mixture, which was diluted with water and filtered. Purified using flash column chromatography on C18 silica (5-95% acetonitrile/water) to give as a white solid (3.506 g, 91.39%). mp > 175 °C. ^{1}H NMR (500 MHz, DMSO-d_6): δ (ppm) 8.08-8.10 (m, 2H), 7.95 (d, *J* = 8.8 Hz, 1H), 7.54-7.63 (m, 3H), 7.33 (d, *J* = 2.2 Hz, 1H), 7.07 (dd, *J* = 2.3, 3.7 Hz, 1H), 6.96 (s, 1H), 4.25 (qui, *J* = 6.1 Hz, 4H), 3.66-3.96 (m, broad peaks, 8H), 3.12-3.30 (m, broad peaks, 16H), 2.14 (qui, *J* = 6.1 Hz, 2H). ^{13}C NMR (125 MHz, DMSO-d_6): δ (ppm) 176.44, 171.36, 169.95, 169.46, 163.00, 162.22, 157.49, 131.70, 131.20, 129.11, 126.28, 126.21, 117.24, 114.94, 106.82, 101.54, 65.30, 61.55, 55.86, 53.20, 53.03, 50.36, 48.85, 27.78. HR-FAB-MS (EI) Anal. Calc. for $C_{34}H_{43}N_4O_{11}$ (MH^+): 683.2928. Found: 683.2925.

Tri-tert-butyl 2,2',2''-(10-(2-(3-((5-hydroxy-4-oxo-2-phenyl-4H-chromen-7-yl)oxy)propoxy)-2-oxoethyl)-1,4,7,10-tetraazacyclododecane-1,4,7-triyl)triacetate (6b). 2,2',2''-(10-(2-(3-((5-hydroxy-4-oxo-2-phenyl-4H-chromen-7-yl)oxy)propoxy)-2-oxoethyl)-1,4,7,10-tetraazacyclododecane-1,4,7-triyl)triacetic acid (7b).

5 (3.440 g, 6.006 mmol) was dissolved in THF (50 mL) and triethylamine (1.275 g, 12.624 mmol) was added. The solution of **1b** (2.748 g, 7.330 mmol) in THF (50 mL) was slowly added and stirred at 40 °C for 2 h. **6b** was dissolved in 1,3-dioxane (20 mL), and HCl (10 mL) was slowly added at 0 °C. The reaction was stirred and slowly allowed to reach 40 °C. After 12 h, removal of the solvent left a crude mixture, which was diluted with water and filtered. Purified using flash column chromatography on C18 silica (5-95% acetonitrile/water) to give as a white solid (3.551 g, 84.74%). mp > 179 °C. ^{1}H NMR (500 MHz, DMSO-d_6): δ (ppm) 12.79 (s, 1H), 8.08 (d, *J* = 7.4 Hz, 2H), 7.56-7.63 (m, 3H), 7.01 (s, 1H), 6.79 (d, *J* = 1.3 Hz, 1H), 6.38 (d, *J* = 1.5 Hz, 1H), 4.23 (t, *J* = 5.8 Hz, 2H), 4.18 (t, *J* = 5.6 Hz, 2H), 3.69-3.71 (m, broad peaks, 8H), 2.90-3.03 (m, broad peaks, 16H), 2.50 (t, *J* = 1.7 Hz, 2H). ^{13}C NMR (125 MHz, DMSO-d_6): δ (ppm)182.07, 171.08, 170.55, 170.52, 164.47, 163.50, 161.22, 157.37, 132.19, 130.60, 129.17, 126.46, 105.38, 105.00, 98.49, 93.27, 65.30, 61.01, 54.27, 53.90, 51.02, 50.42, 49.64, 49.31, 27.82. HR-FAB-MS (EI) Anal. Calc. for $C_{34}H_{43}N_4O_{12}$ (MH^+): 699.2877. Found: 699.2881.

Tri-tert-butyl 2,2',2''-(10-(2-(3-((3,5-diacetoxy-4-oxo-2-phenyl-4H-chromen-7-yl)oxy)propoxy)-2-oxoethyl)-1,4,7,10-tetraazacyclododecane-1,4,7-triyl)triacetate (6c). 2,2',2''-(10-(2-(3-((3,5-Dihydroxy-4-oxo-2-phenyl-4H-chromen-7-yl)oxy)propoxy)-2-oxoethyl)-1,4,7,10-tetraazacyclododecane-1,4,7-triyl)triacetic acid (7c).

5 (3.028 g, 5.286 mmol) was dissolved in THF (50 mL) and triethylamine (1.605 g, 15.8583 mmol) was added. The solution of **1c** (3.014 g, 6.3433 mmol) in THF (50 mL) was slowly added and stirred at 40 °C for 2 h. **6c** was dissolved in 1,3-dioxane (20 mL), and HCl (10 mL) was slowly added at 0 °C. The reaction was stirred and slowly allowed to reach 40 °C. After 12 h, removal of the solvent left a crude mixture, which was diluted with water and filtered. Purified using flash column chromatography on C18 silica (5-95% acetonitrile/water) to give as a light yellow solid (2.647 g, 70.11%). mp > 230 °C. ^{1}H NMR (500 MHz, DMSO-d_6): δ (ppm) 12.33 (s, 1H), 9.81 (s, broad peak, 1H), 8.17-8.19 (m, 2H), 7.48-7.57 (m, 3H), 6.75 (d, *J* = 2.1 Hz, 1H), 6.35 (d, *J* = 2.1 Hz, 1H), 4.23 (t, *J* = 6.3 Hz, 2H), 4.17 (t, *J* = 6.1 Hz, 2H), 3.66-3.71 (m, broad peaks, 8H), 2.90-3.07 (m, broad peaks, 16H), 2.08 (qui, *J* = 6.2 Hz, 2H). ^{13}C NMR (125 MHz, DMSO-d_6): δ (ppm) 176.46, 171.11, 170.59, 170.53, 164.28, 160.48, 156.34, 146.16, 137.49, 130.91, 130.10, 128.56, 127.62, 104.29, 97.89, 92.58, 65.26, 61.02, 54.29, 53.92, 53.88, 51.03, 50.43, 49.63, 49.29, 27.84. HR-FAB-MS (EI) Anal. Calc. for $C_{34}H_{43}N_4O_{13}$ (MH^+): 715.2827. Found: 715.2829.

General procedure for the synthesis of 8.

7 (1 eq) and $GdCl_3{\cdot}6H_2O$ (1-1.2 eq) was dissolved in deionized water and pH of reaction mixture was adjusted to 6 with 1 M $NaHCO_3$ solution. The mixture was stirred at room temperature and monitored by TLC and LC-MS. The mixture was centrifuged at 5,000 rpm for 10 minutes. The supernatant was filtered with a 0.25 μm filter. Purified using flash column chromatography on C18 silica (5-95% acetonitrile/water) to give as **8a-c**.

Gd-flavone (8a).

7a (1.025 g, 1.501 mmol, 1 eq) and $GdCl_3{\cdot}6H_2O$ (0.558 g, 1.505 mmol, 1 eq) was dissolved in deionized water and pH of reaction mixture was adjusted to 6 with 1 M $NaHCO_3$ solution. White solid (1.076 g, 85.69%). The purity was confirmed by analytical HPLC (Figure S28, 96.00%). mp > 216 °C. HR-FAB-MS (EI) Anal. Calc. for $C_{34}H_{40}GdN_4O_{11}$ (MH^+): 838.1935. Found: 838.1938.

Gd-chrysin (8b).

7b (1.047g, 1.498 mmol) and $GdCl_3{\cdot}6H_2O$ (0.558 g, 1.506 mmol, 1 eq) was dissolved in deionized water and pH of reaction mixture was adjusted to 6 with 1 M $NaHCO_3$ solution. White solid (1.079 g, 84.32%). The purity was confirmed by analytical HPLC (Figure S30, 95.85%). mp > 250 °C. HR-FAB-MS (EI) Anal. Calc. for $C_{34}H_{40}GdN_4O_{12}$ (MH^+): 854.1884. Found: 854.1888.

Gd-galangin (8c).

7c (1 eq) and $GdCl_3$ $6H_2O$ (1.2 eq) was dissolved in deionized water and pH of reaction mixture was adjusted to 6 with 1 M NaHCO3 solution. Yellow solid (44.41-67.95%). The purity was confirmed by analytical HPLC (Figure S32, 97.50%). mp > 225 °C. HR-FAB-MS (EI) Anal. Calc. for $C_{34}H_{40}N_4GdO_{13}$ (MH^+): 870.1833. Found: 870.1837.

4-Oxo-2-phenyl-4H-chromene-3,5,7-triyl triacetate (2).

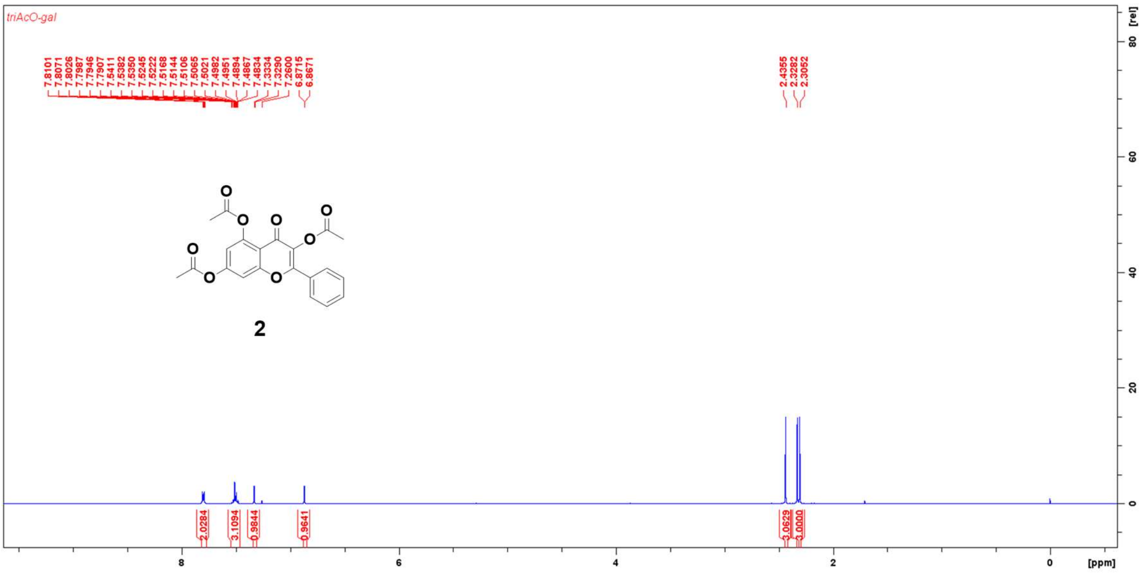

Figure S1. ^{1}H NMR spectrum of compound **2.**

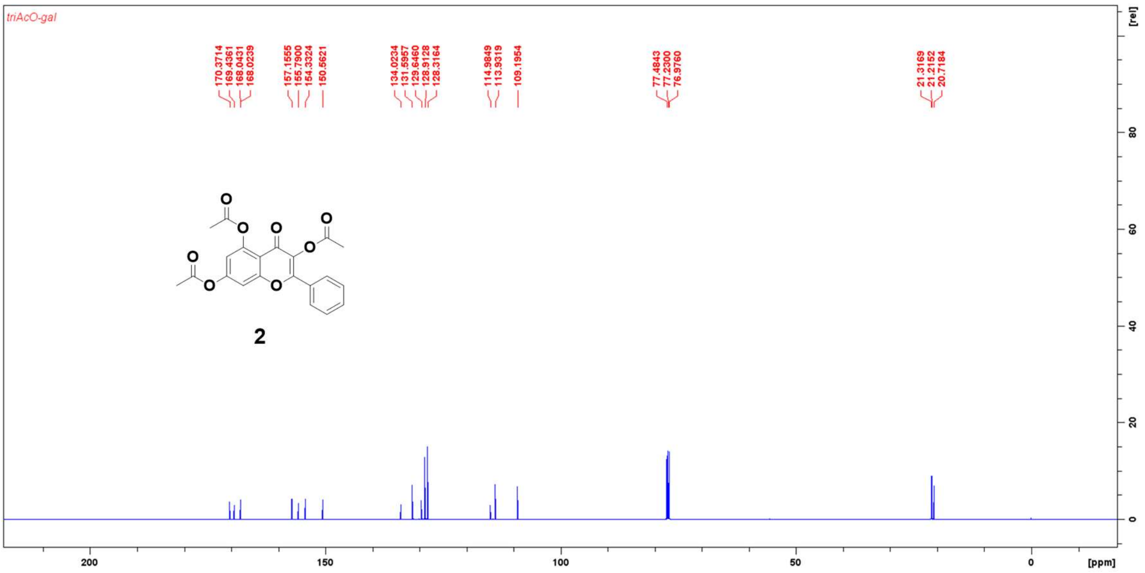

Figure S2. ^{13}C NMR spectrum of compound **2.**

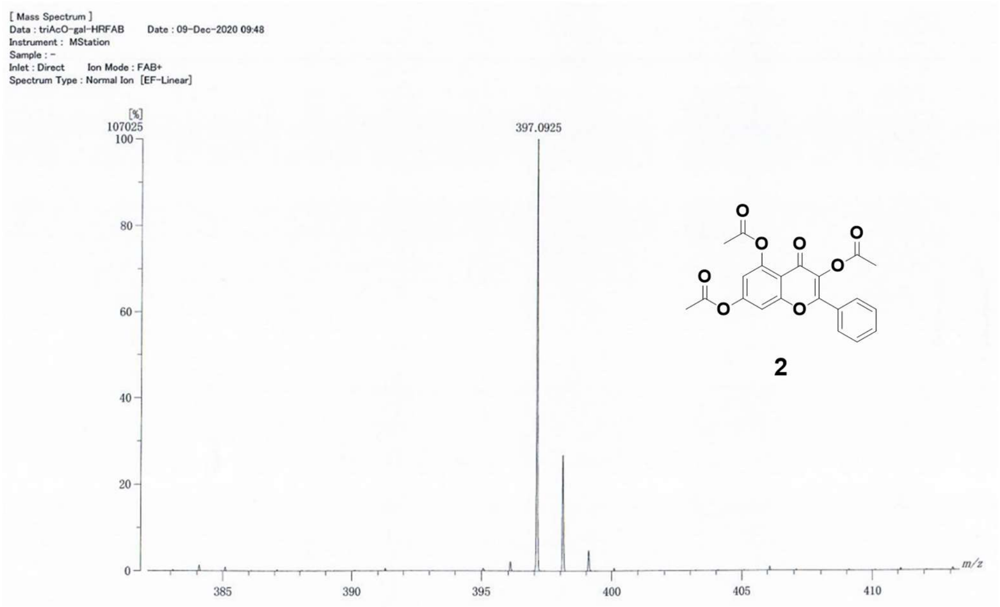

Figure S3. High resolution FAB-mass spectrum of compound **2.**

7-Hydroxy-4-oxo-2-phenyl-4H-chromene-3,5-diyl diacetate (3).

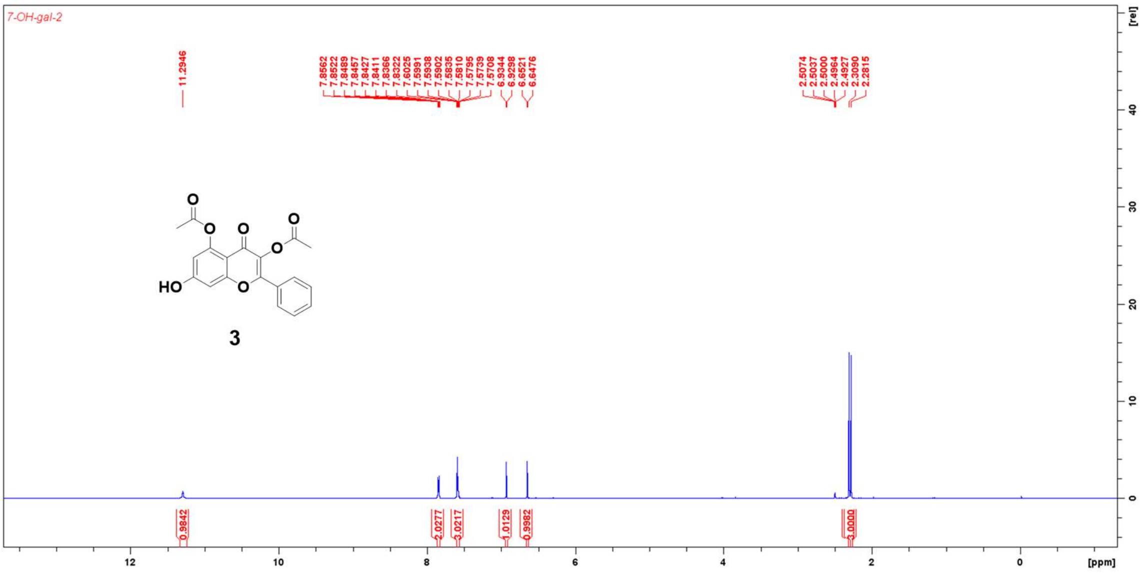

Figure S4. ^{1}H NMR spectrum of compound **3.**

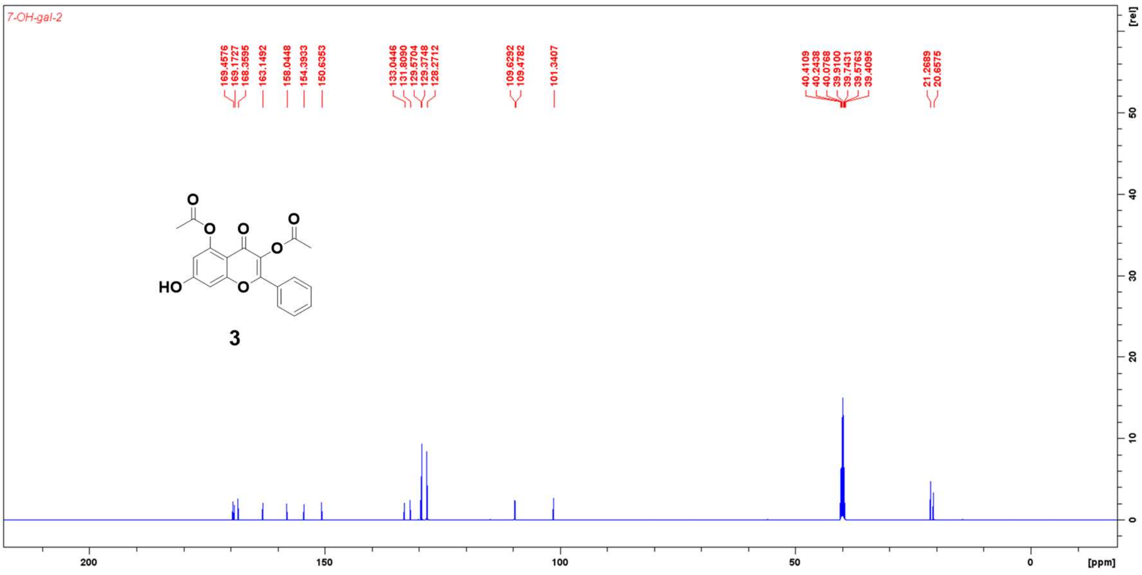

Figure S5. ^{13}C NMR spectrum of compound **3.**

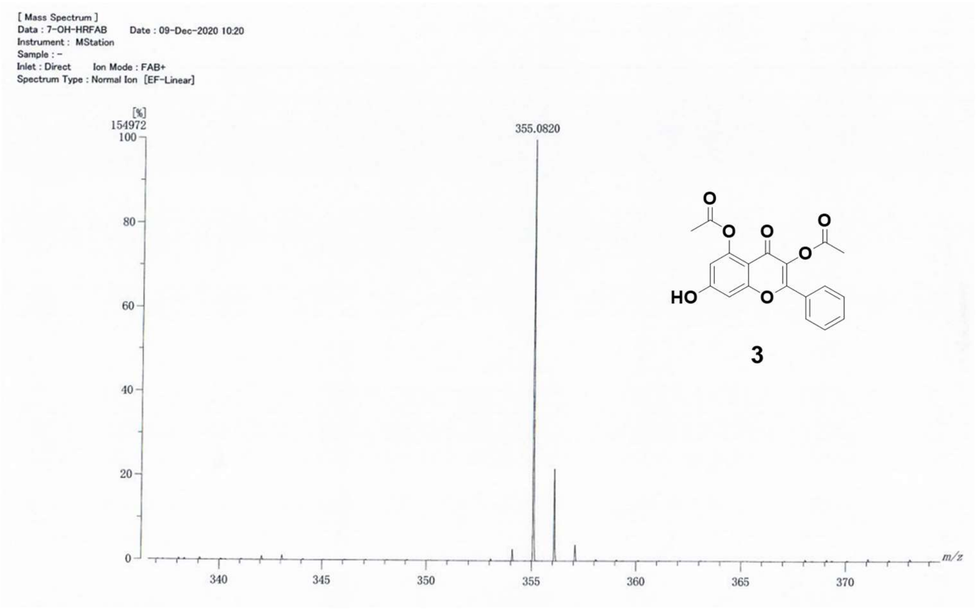

Figure S6. High resolution FAB-mass spectrum of compound **3.**

7-(3-Bromopropoxy)-2-phenyl-4H-chromen-4-one (1a).

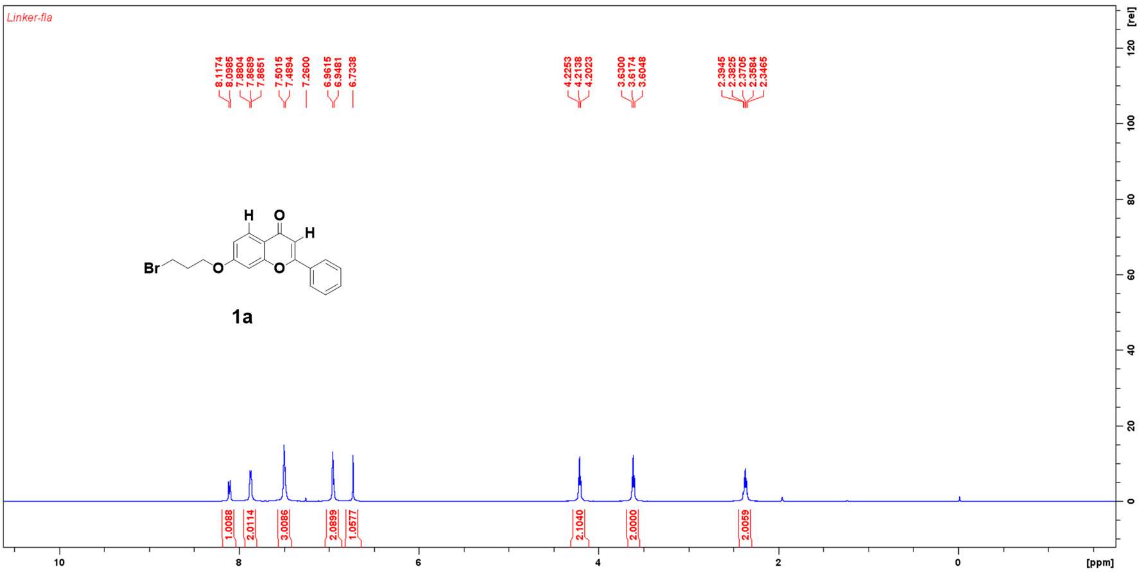

Figure S7. ^{1}H NMR spectrum of compound **1a.**

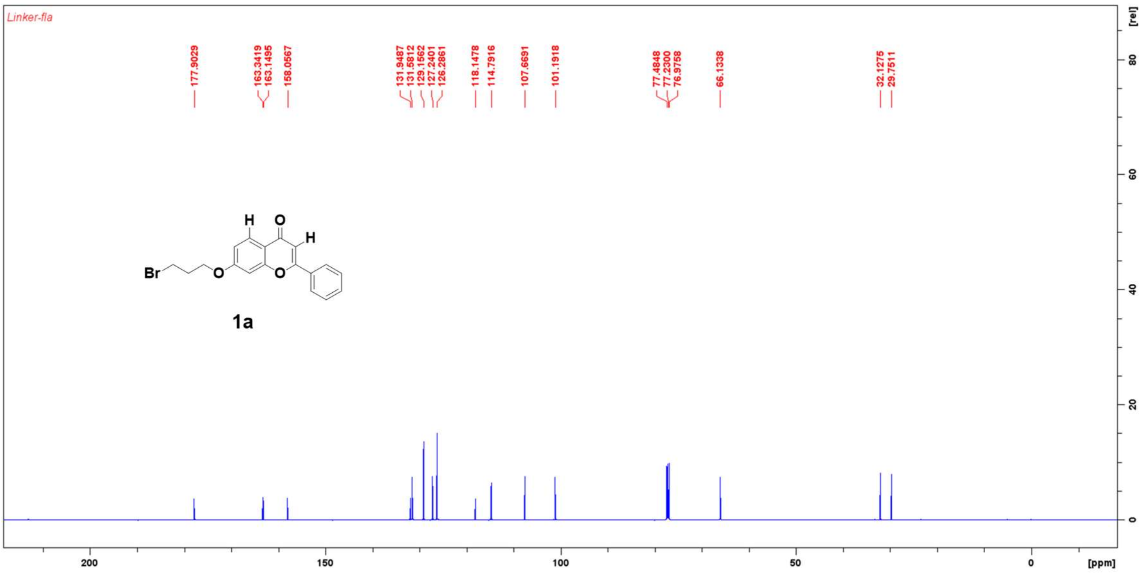

Figure S8. ^{13}C NMR spectrum of compound **1a.**

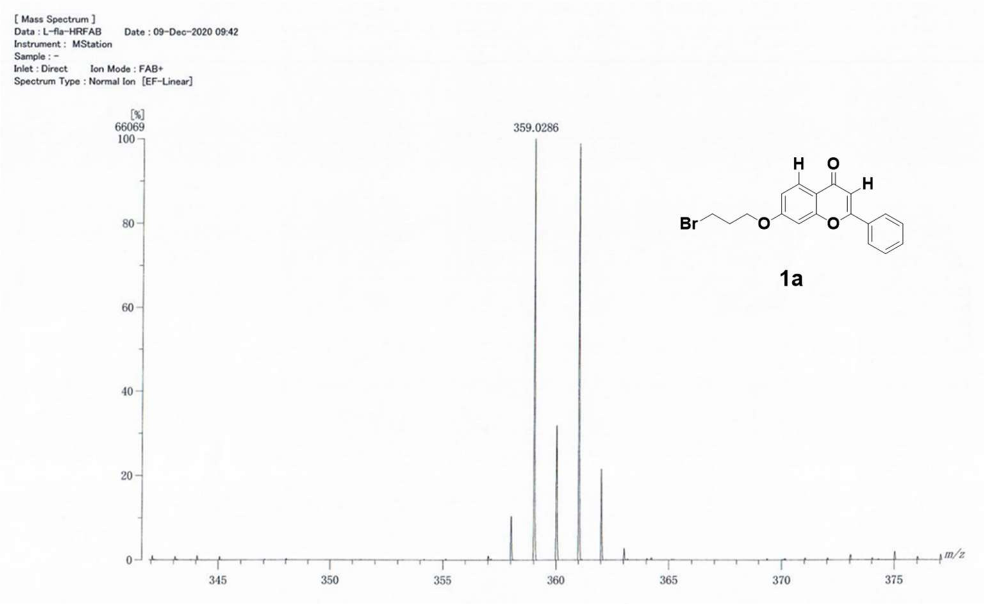

Figure S9. High resolution FAB-mass spectrum of compound **1a.**

7-(3-Bromopropoxy)-5-hydroxy-2-phenyl-4H-chromen-4-one (1b).

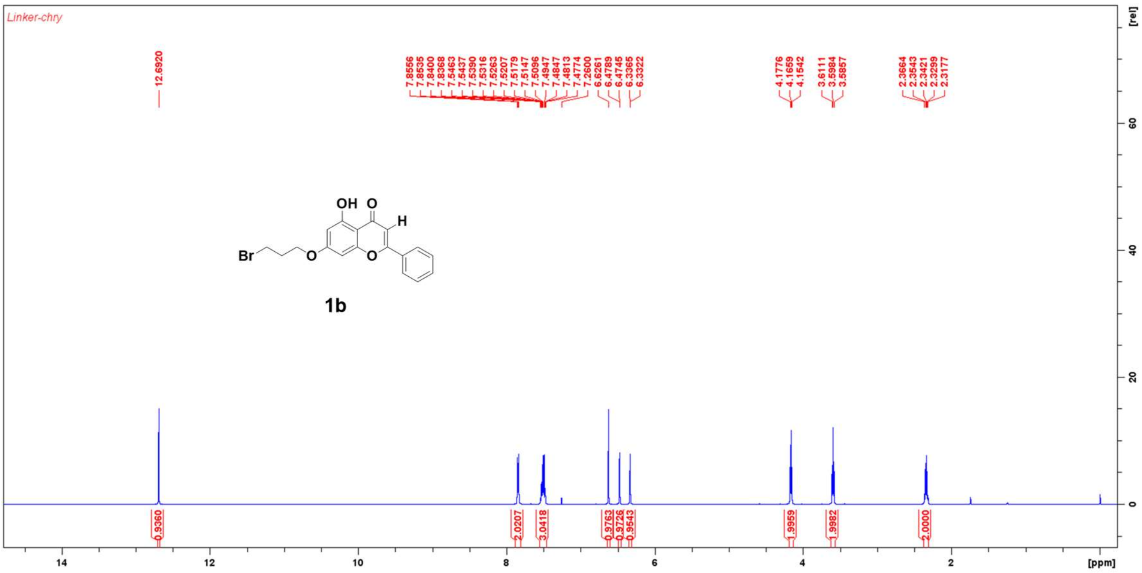

Figure S10. ^{1}H NMR spectrum of compound **1b.**

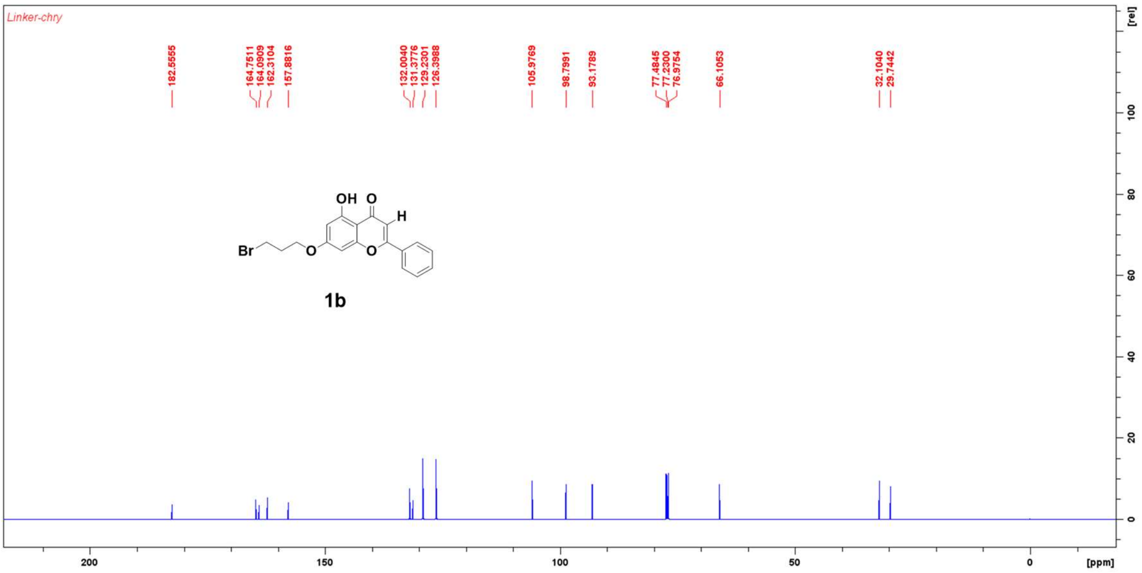

Figure S11. ^{13}C NMR spectrum of compound **1b.**

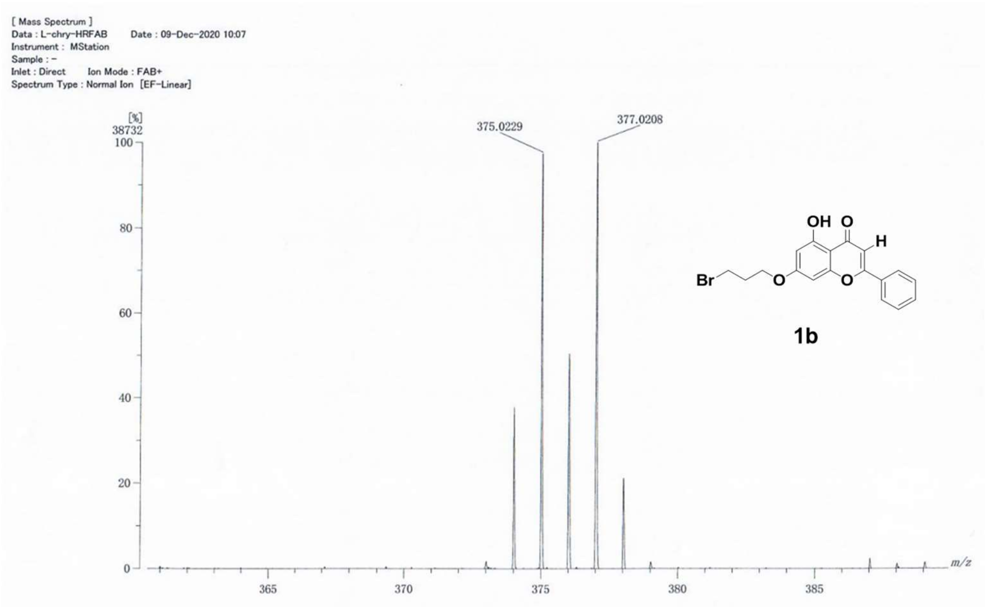

Figure S12. High resolution FAB-mass spectrum of compound **1b.**

7-(3-Bromopropoxy)-4-oxo-2-phenyl-4H-chromene-3,5-diyl diacetate (1c).

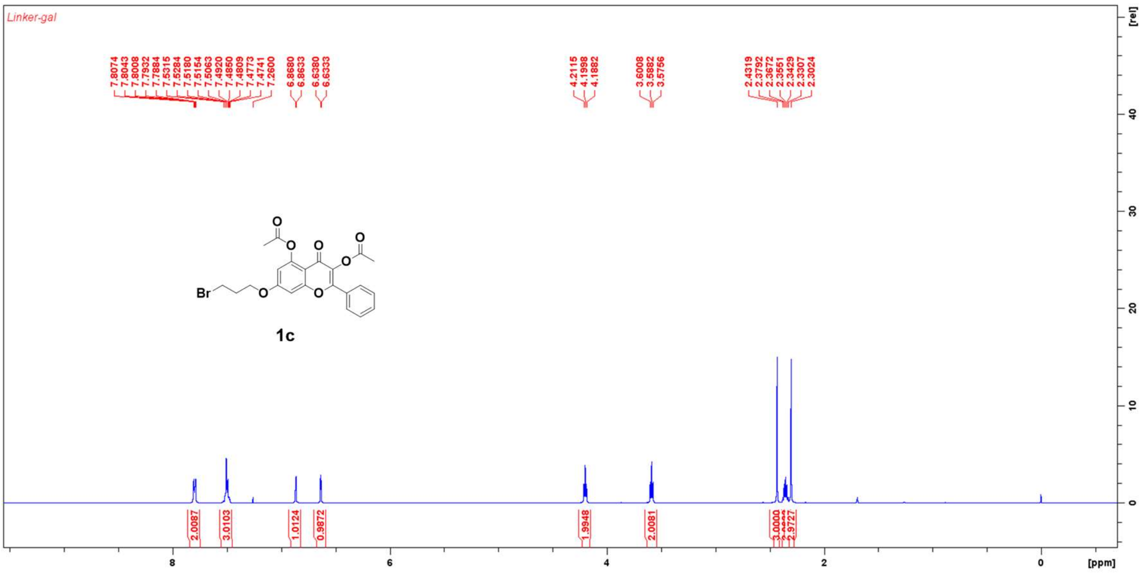

Figure S13. ^{1}H NMR spectrum of compound **1c.**

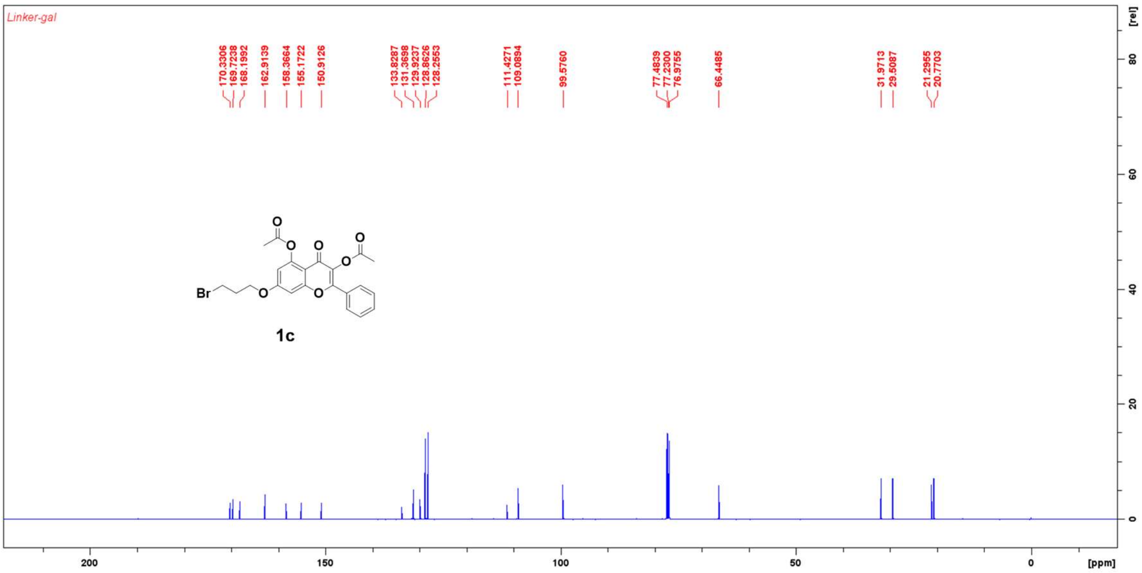

Figure S14. ^{13}C NMR spectrum of compound **1c.**

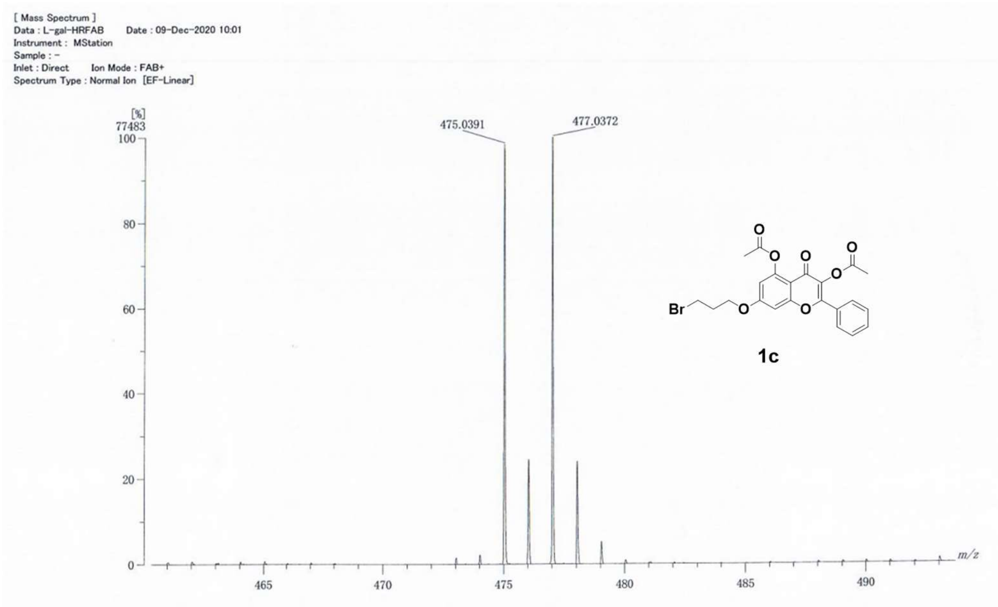

Figure S15. High resolution FAB-mass spectrum of compound **1c.**

2-(4,7,10-Tris(2-(tert-butoxy)-2-oxoethyl)-1,4,7,10-tetraazacyclododecan-1-yl)acetic acid (5).

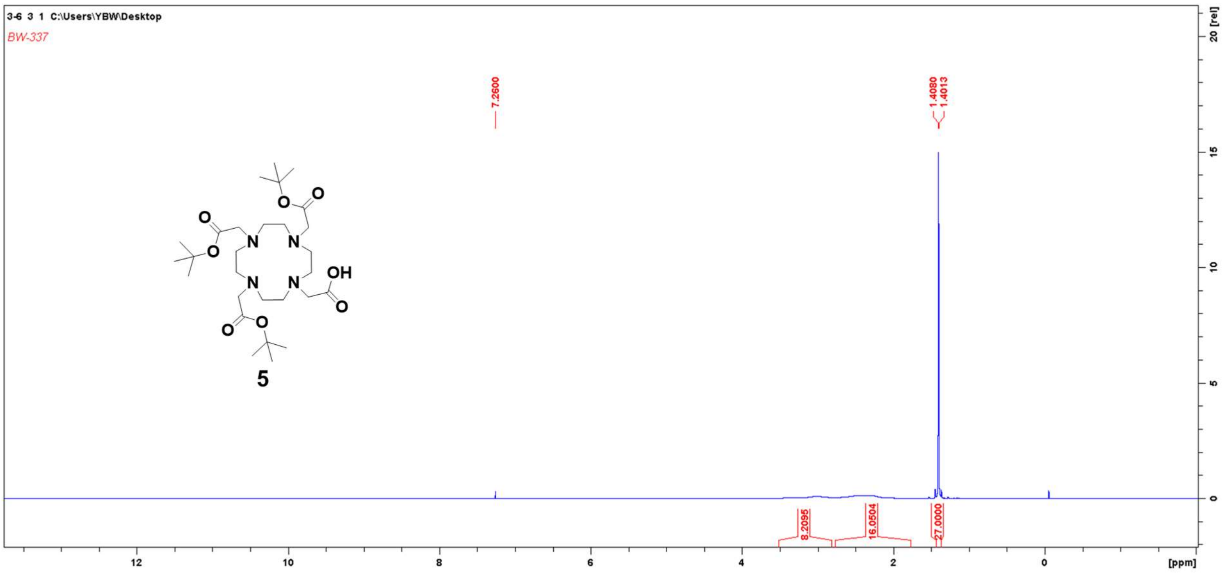

Figure S16. ^{1}H NMR spectrum of compound **5.**

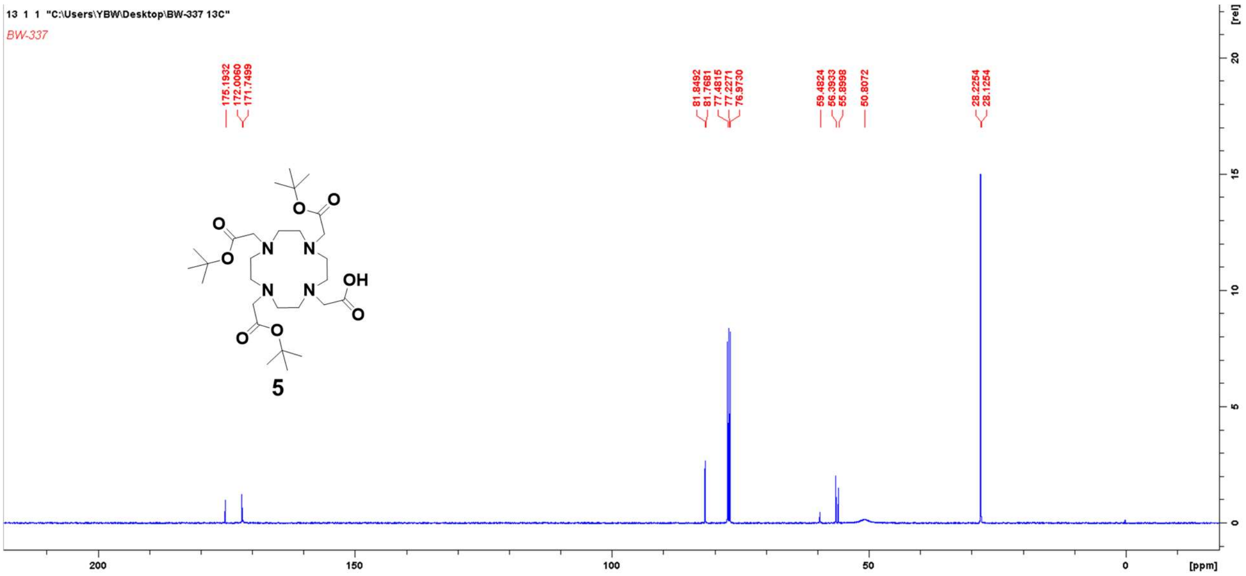

Figure S17. ^{13}C NMR spectrum of compound **5.**

2,2',2''-(10-(2-Oxo-2-(3-((4-oxo-2-phenyl-4H-chromen-7-yl)oxy)propoxy)ethyl)-1,4,7,10-tetraazacyclododecane-1,4,7-triyl)triacetic acid (7a).

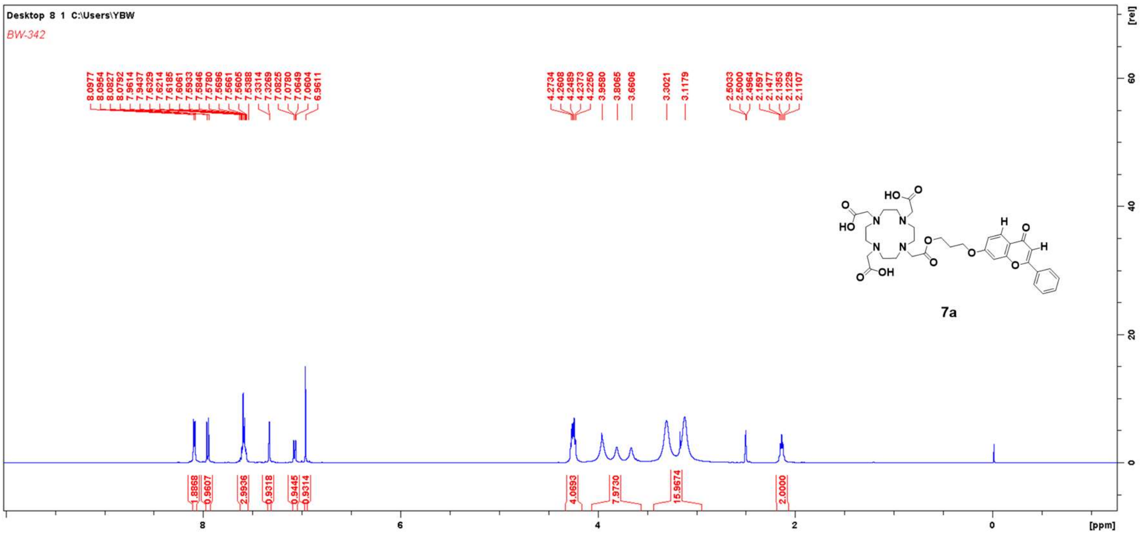

Figure S18. ^{1}H NMR spectrum of compound **7a.**

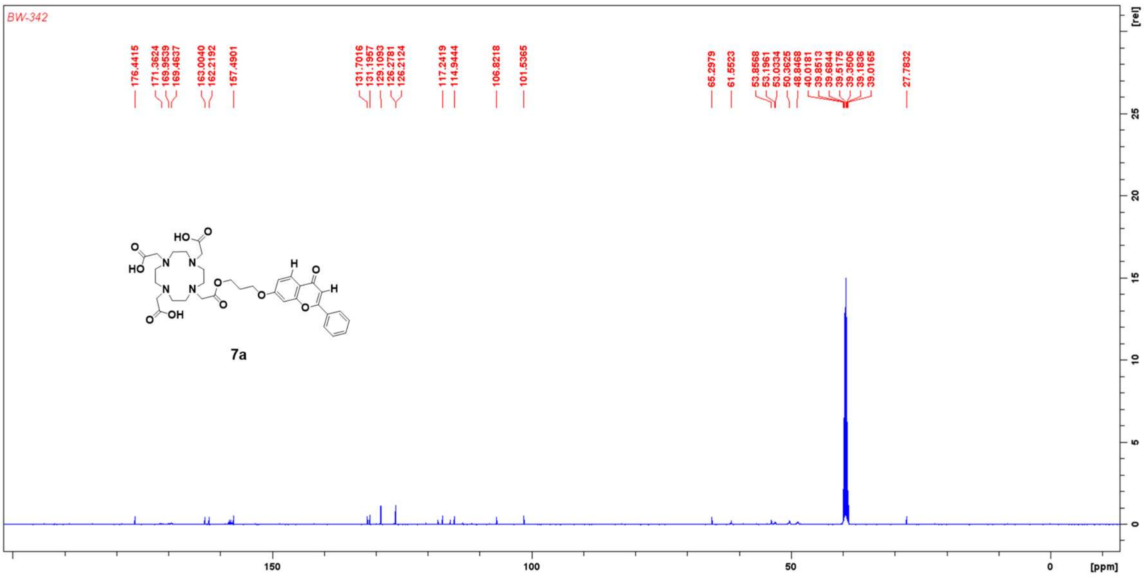

Figure S19. ^{13}C NMR spectrum of compound **7a.**

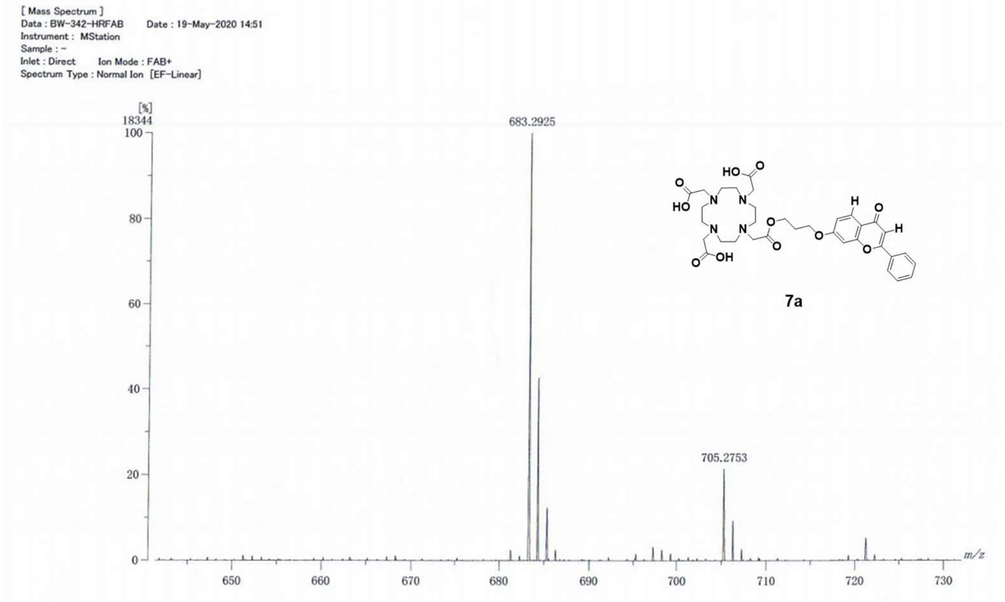

Figure S20. High resolution FAB-mass spectrum of compound **7a.**

2,2',2''-(10-(2-(3-((5-hydroxy-4-oxo-2-phenyl-4H-chromen-7-yl)oxy)propoxy)-2-oxoethyl)-1,4,7,10-tetraazacyclododecane-1,4,7-triyl)triacetic acid (7b).

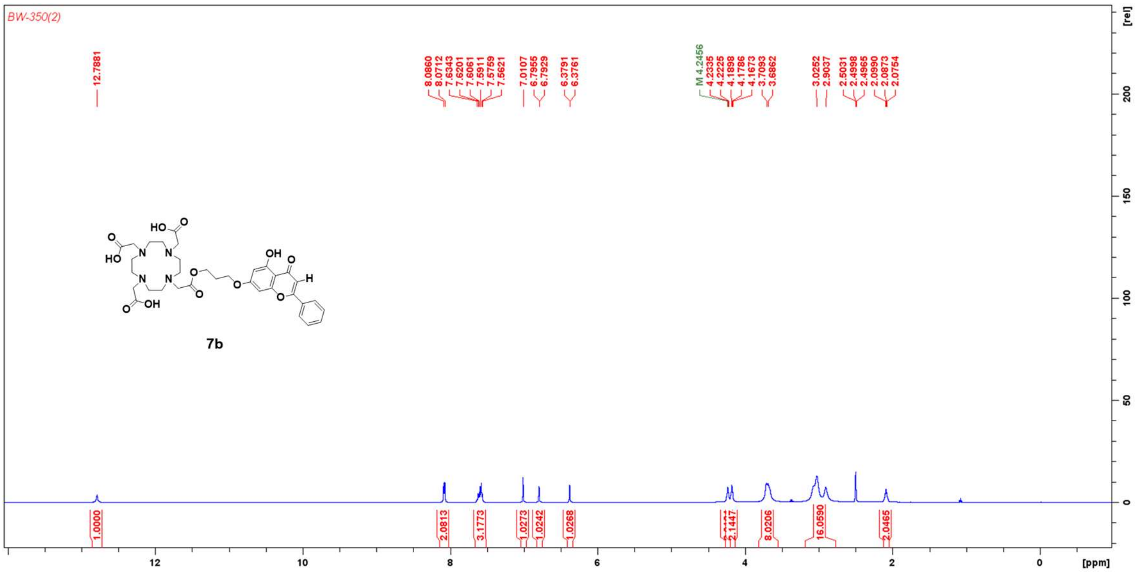

Figure S21. ^{1}H NMR spectrum of compound **7b.**

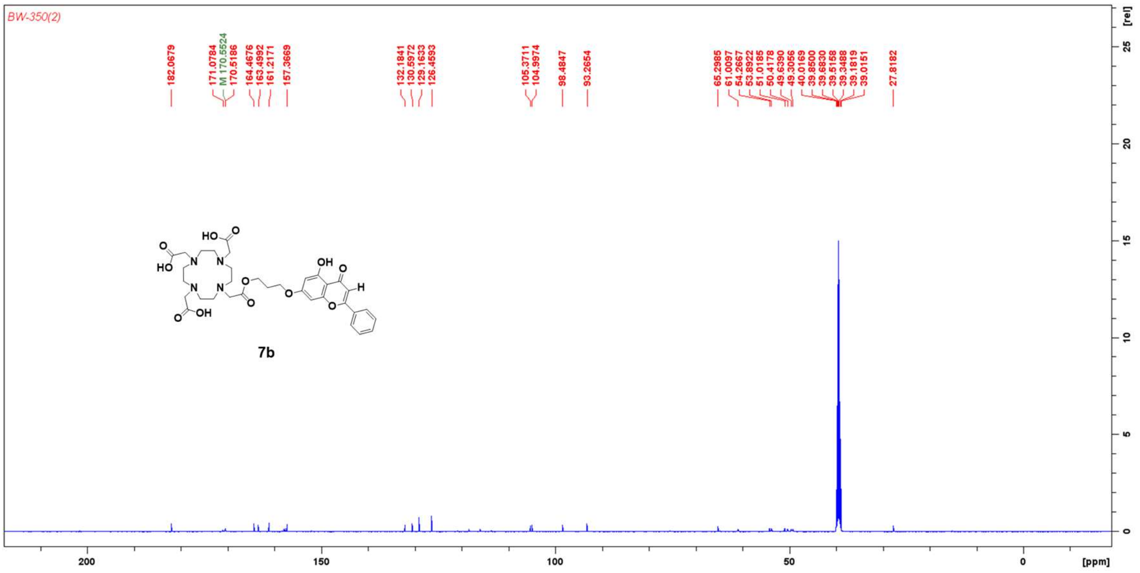

Figure S22. ^{13}C NMR spectrum of compound **7b.**

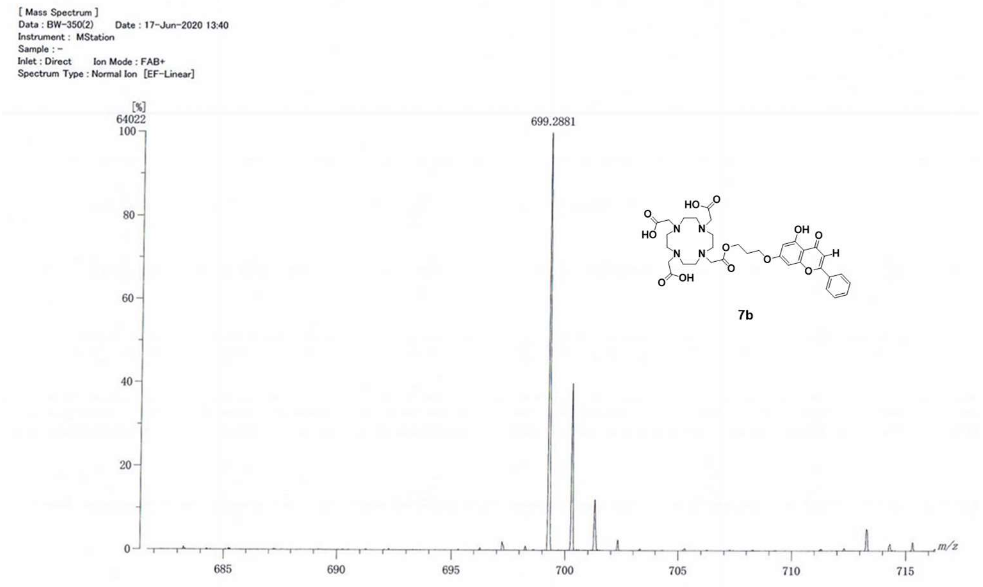

Figure S23. High resolution FAB-mass spectrum of compound **7b.**

2,2',2''-(10-(2-(3-((3,5-Dihydroxy-4-oxo-2-phenyl-4H-chromen-7-yl)oxy)propoxy)-2-oxoethyl)-1,4,7,10-tetraazacyclododecane-1,4,7-triyl)triacetic acid (7c).

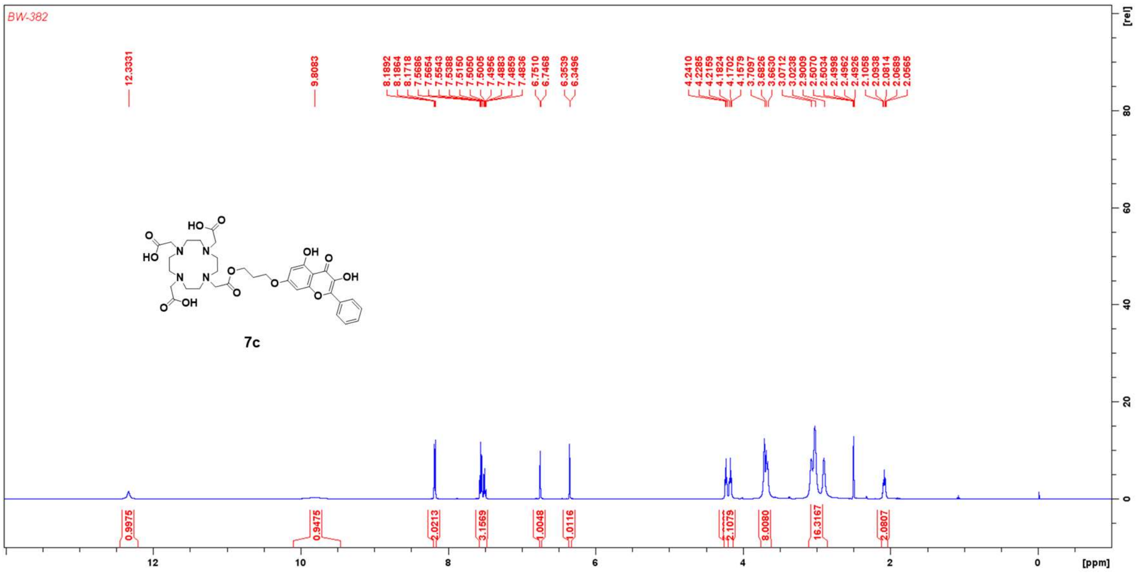

Figure S24. ^{1}H NMR spectrum of compound **7c.**

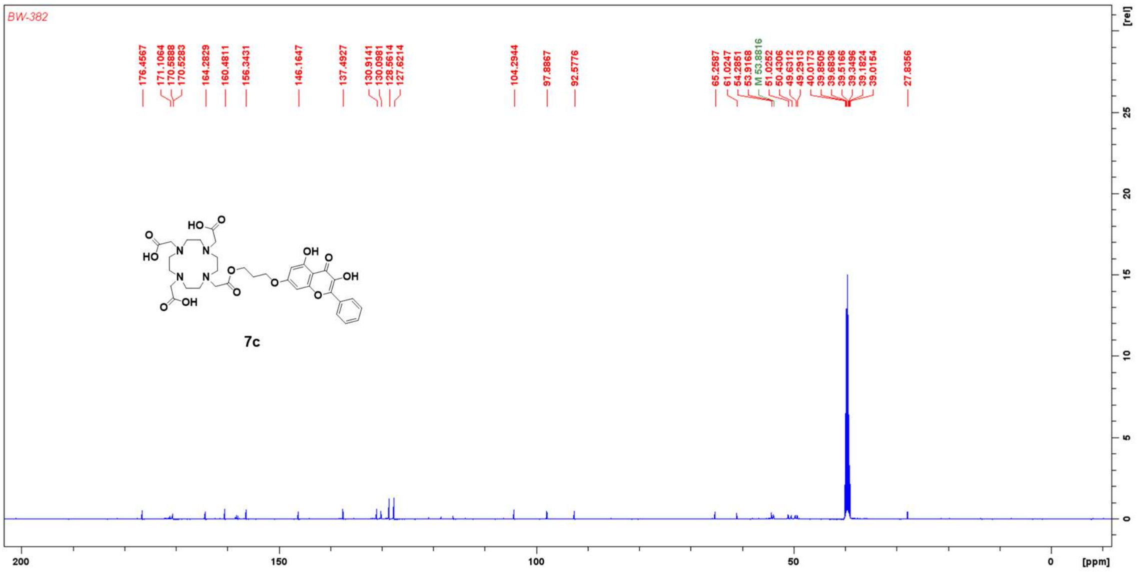

Figure S25. ^{13}C NMR spectrum of compound **7c.**

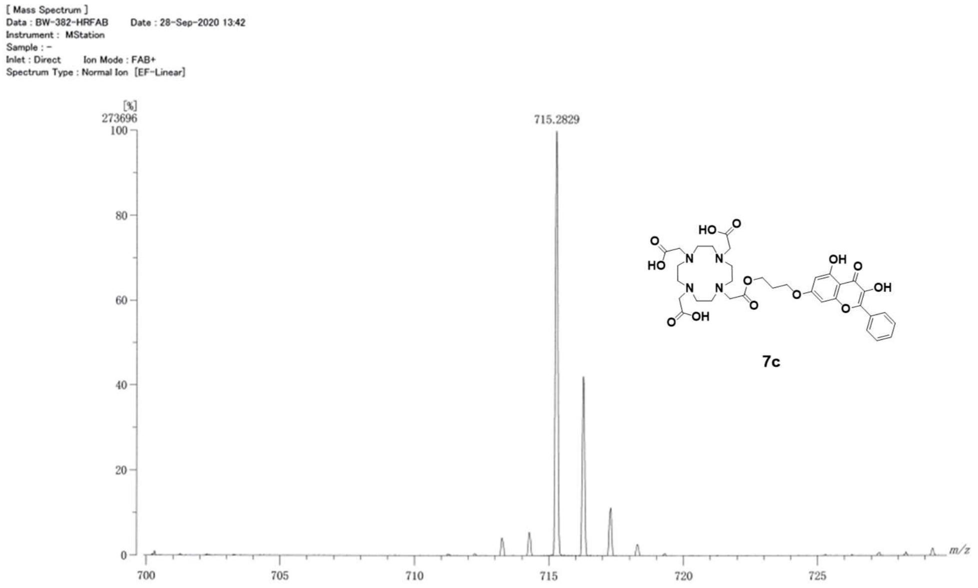

Figure S26. High resolution FAB-mass spectrum of compound **7c.**

Gd-flavone (8a).

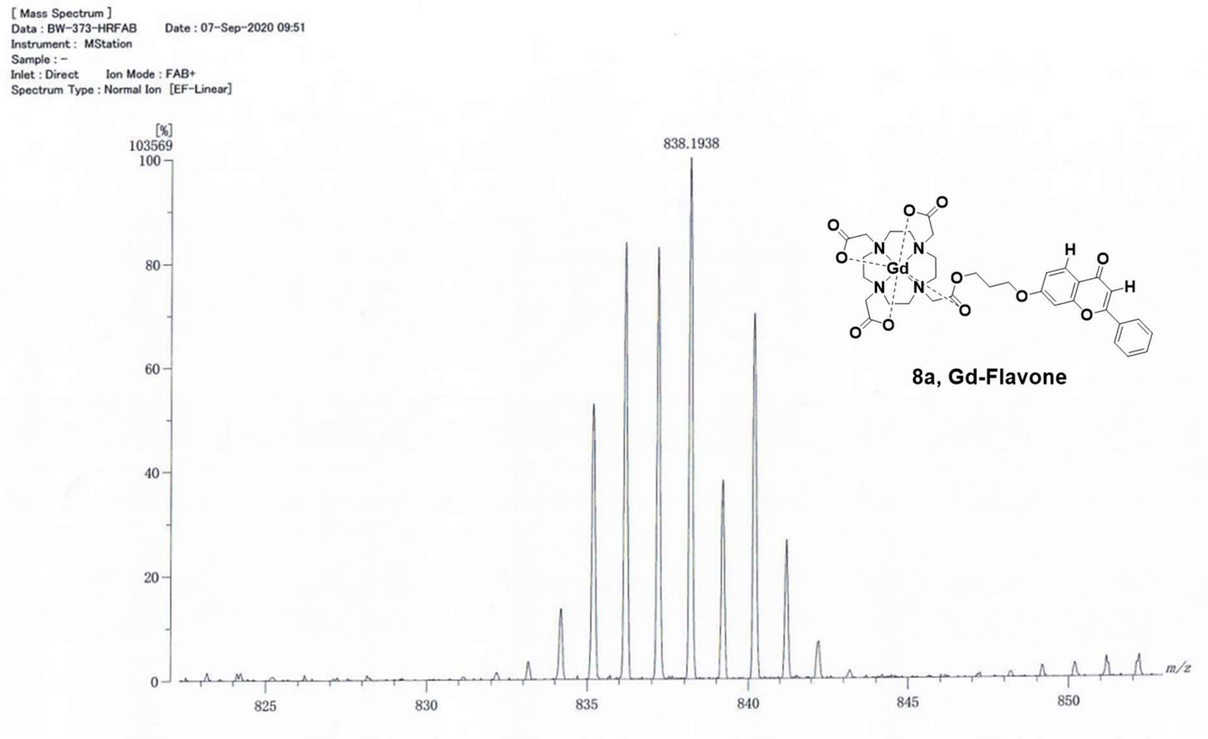

Figure S27. High resolution FAB-mass spectrum of compound **8a.**

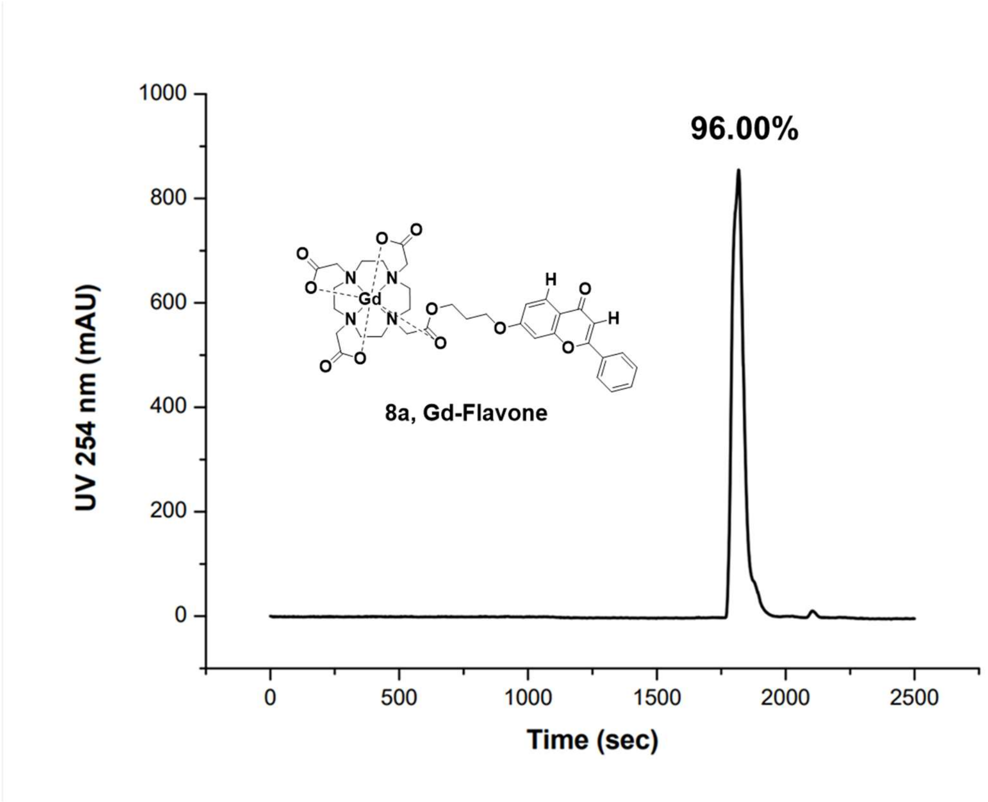

Figure S28. HPLC spectrum of compound **8a**. The peak area represents each purity of compounds

Gd-chrysin (8b).

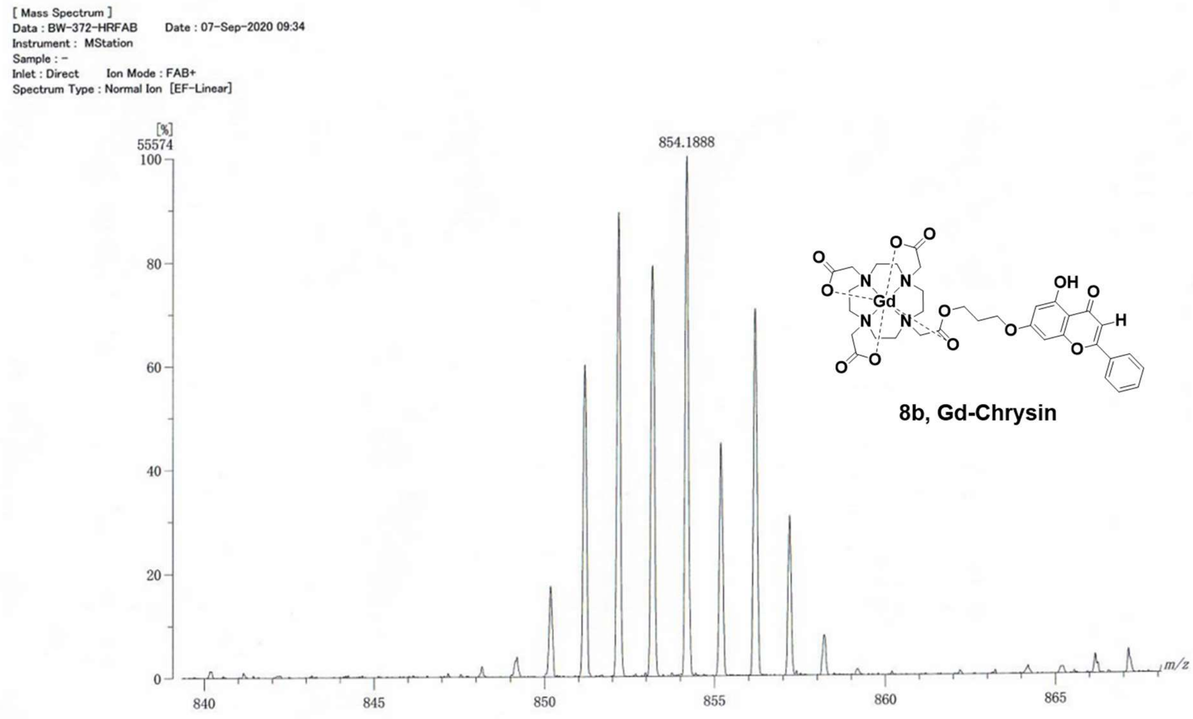

Figure S29. High resolution FAB-mass spectrum of compound **8b.**

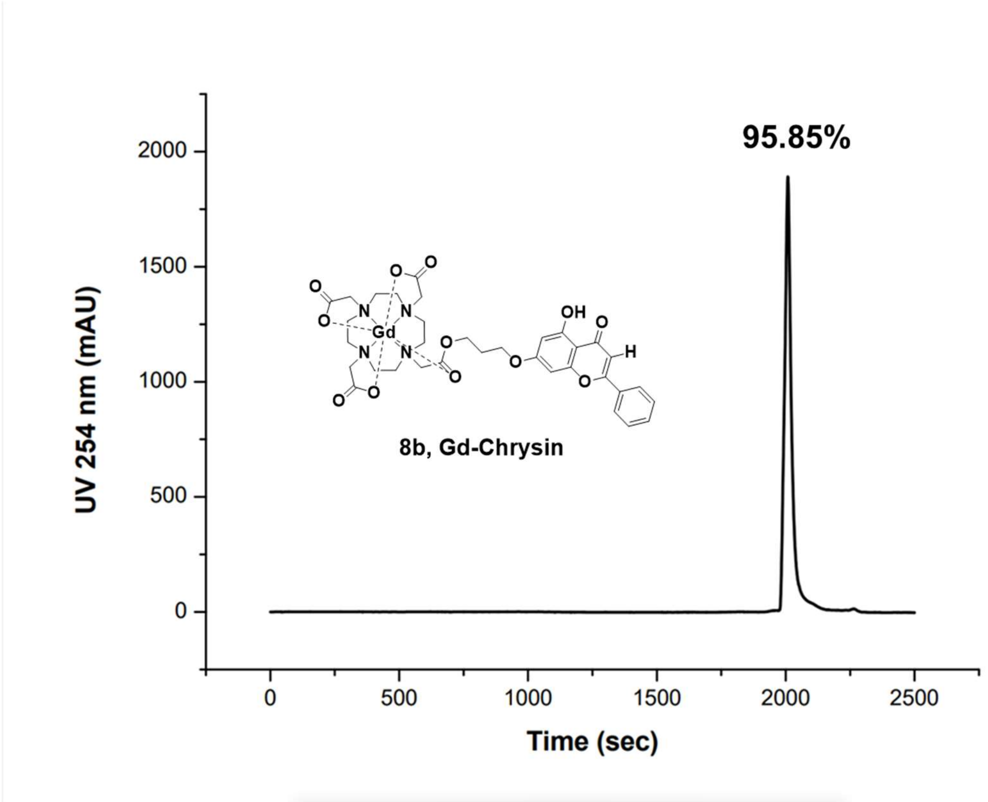

Figure S30. HPLC spectrum of compound **8b**. The peak area represents each purity of compounds

Gd-galangin (8c).

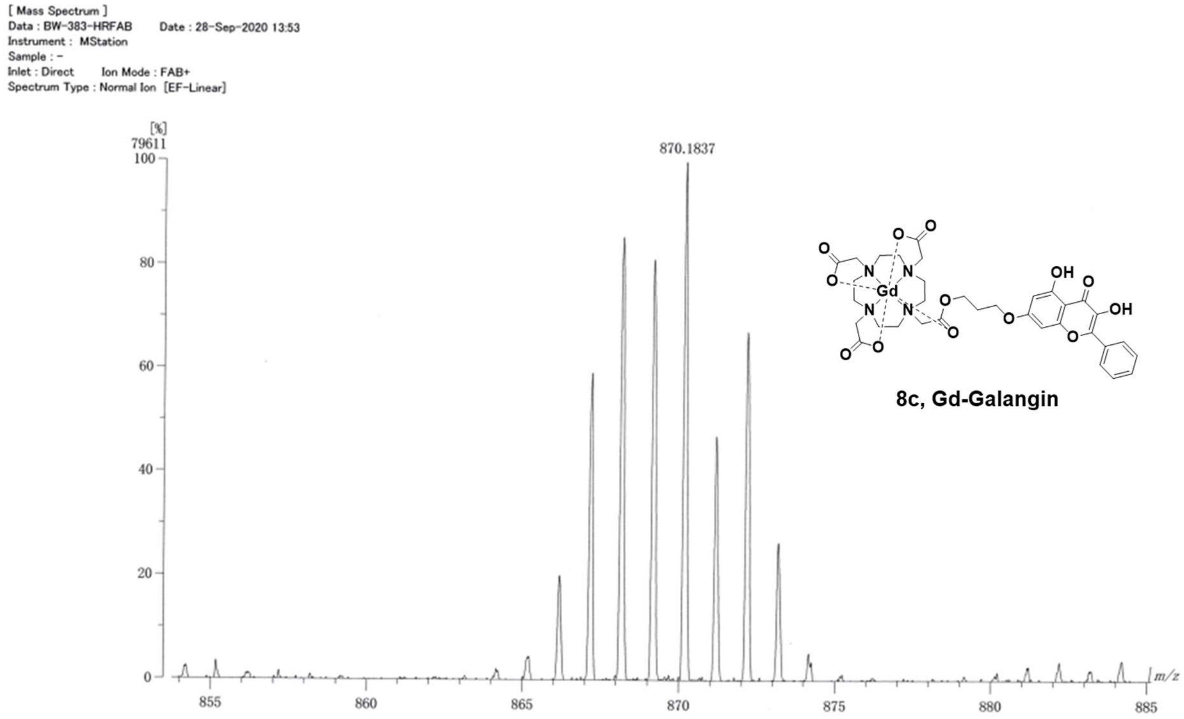

Figure 31. High resolution FAB-mass spectrum of compound **8c.**

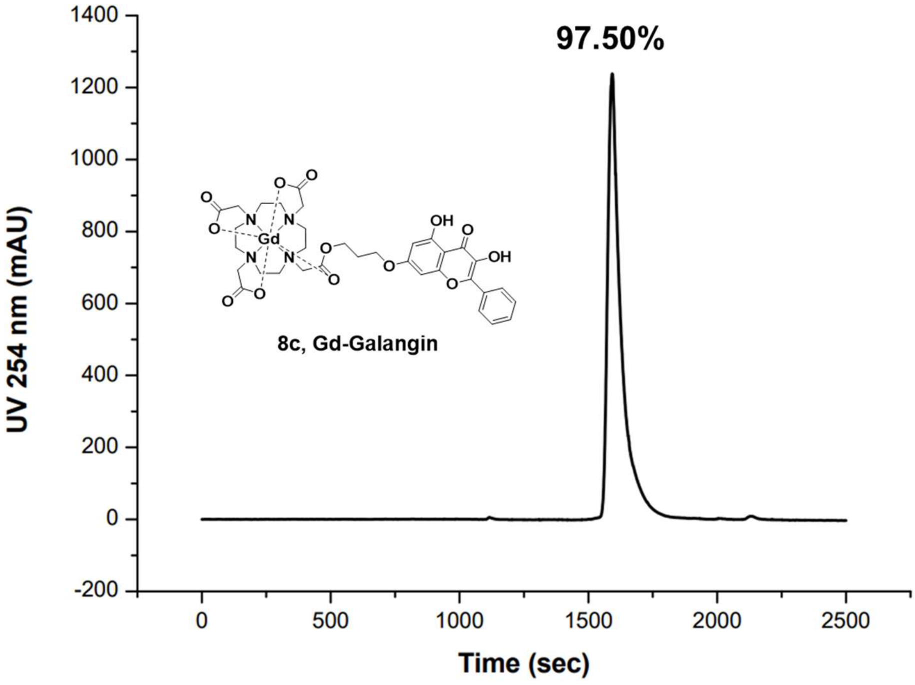

Figure S32. HPLC spectrum of compound **8c**. The peak area represents each purity of compounds

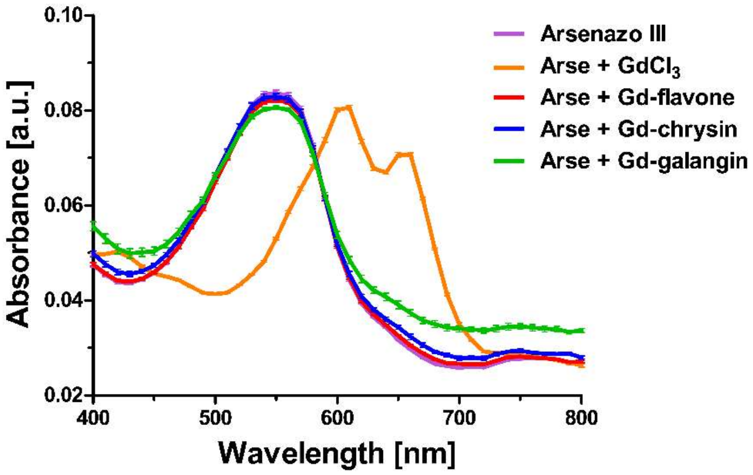

Figure S33. Absorbance spectra for free gadolinium verification using arsenazo III

2. Radical Scavenging Study

2.1. 2,2-Diphenyl-1-picrylhydrazyl (DPPH) Radical Scavenging Activity

The free radical scavenging activities of the synthesized materials were confirmed based on the bleaching of the 2,2-diphenyl-1-picrylhydrazyl (DPPH) radical. Briefly, several concentrations were prepared and mixed with an ethanol solution of DPPH. Thereafter, the samples were thoroughly mixed and incubated for 30 min in the dark at room temperature. Absorbance was measured at 525 nm by employing a SpectraMax i3 microplate reader (Molecular Devices, CA, USA). The antioxidant effect was determined in terms of the DPPH radical removal rate calculated using the following equation:

$$\%\ DPPH\ radical\ scavenging = Control\ Absorbance - Sample\ AbsorbanceControl\ Absorbance \times 100 \quad (1)$$

As a control, we measured the absorbance of the DPPH solution, and the sample absorbance was that of the DPPH solution measured after sample addition [67]. EC_{50} values were determined from a graph showing the percentage inhibition at different concentrations. Analysis was performed using GraphPad Prism version 5.03 (GraphPad Prism software Inc., San Diego, CA, USA).

2.2. Ferric Reducing Antioxidant Potential (FRAP) Radical Scavenging Activity

Another radical scavenging activities of the synthesized Gd complexes were confirmed by changing the color of the FRAP solution. Briefly, several concentrations were prepared, and Gd complexes mixed with FRAP reagent. And then, samples were incubated for 30 min at 37 °C. The absorbance was obtained at 594 nm using a microplate reader system. The antioxidant effect on FRAP can be known from the following equation:

$$FRAP\ value = c\ \times V\ \times t/m \quad (2)$$

Where c is the concentration of the corresponding Trolox, V is the sample volume, t is the dilution factor, and m is the sample weight [68]. Analysis was performed using GraphPad Prism.

2.3. 2,2′-Azino-bis (3-ethylbenzthiazoline-6-sulphonic acid (ABTS) Radical Scavenging Activity

The radical scavenging activities of the synthesized materials were also assessed using a 2,2'-azino-bis (3-ethylbenzthiazoline-6-sulphonic acid) (ABTS) reagent. Briefly, samples were prepared and mixed with ABTS reagent and potassium peroxydisulfate followed by incubation for 16 h at 37 °C in the dark. Absorbance was measured at 734 nm using a SpectraMax i3 microplate reader and the antioxidant effect on ABTS was determined using the following equation:

$$\%\ ABTS\ scavenging\ activity\ = (1 - A/Ao) \times\ 100 \quad (3)$$

where A_0 is the absorbance of the negative control, and A is the absorbance of the sample mixture. Trolox and BHT, with final concentrations in the range of 0.67–2.67 μg/mL, were prepared as positive controls [69]. The analysis was conducted using GraphPad Prism.

3. Other Figures

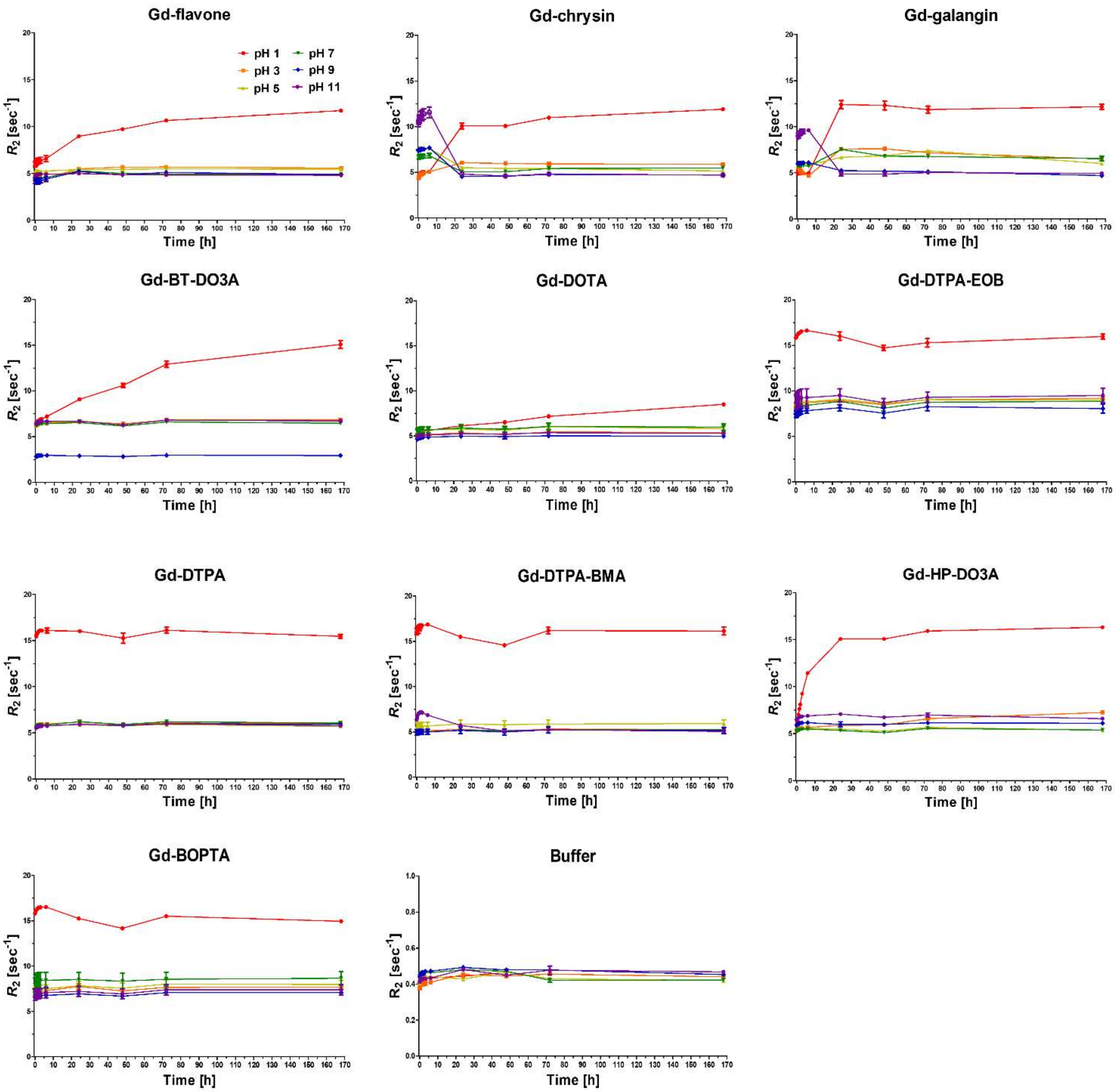

Figure S34. pH stability of Gd-flavone, Gd-chrysin, Gd-galangin and commercial magnetic resonance (MR) contrast agents. Samples prepared at the same concentration with various pH buffers were measured at 3.0 T for 7 days.

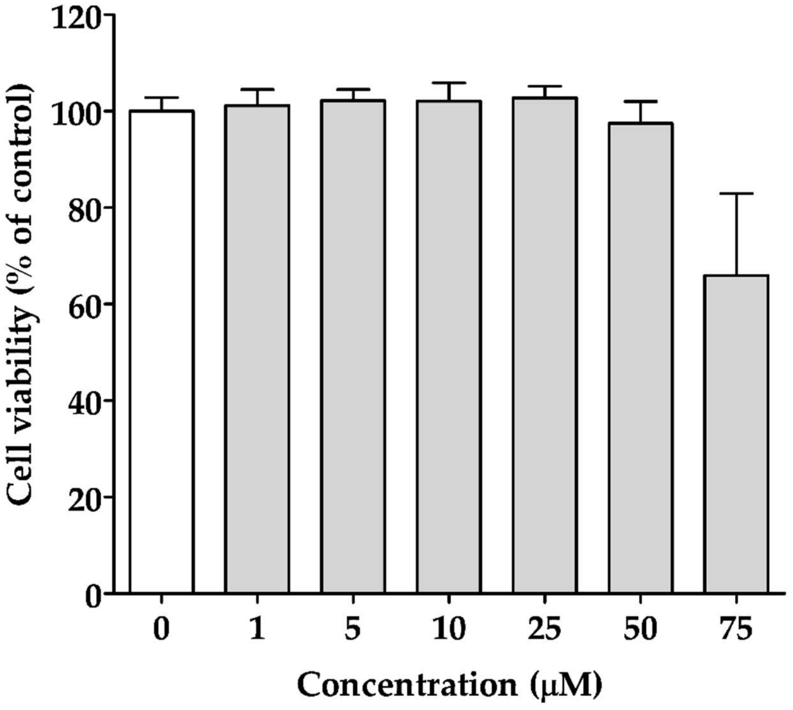

Figure S35. Cell viability of RAW 264.7 mouse macrophage cell in various concentration of Gd-galangin. Expected IC 50 value 81.99 μM

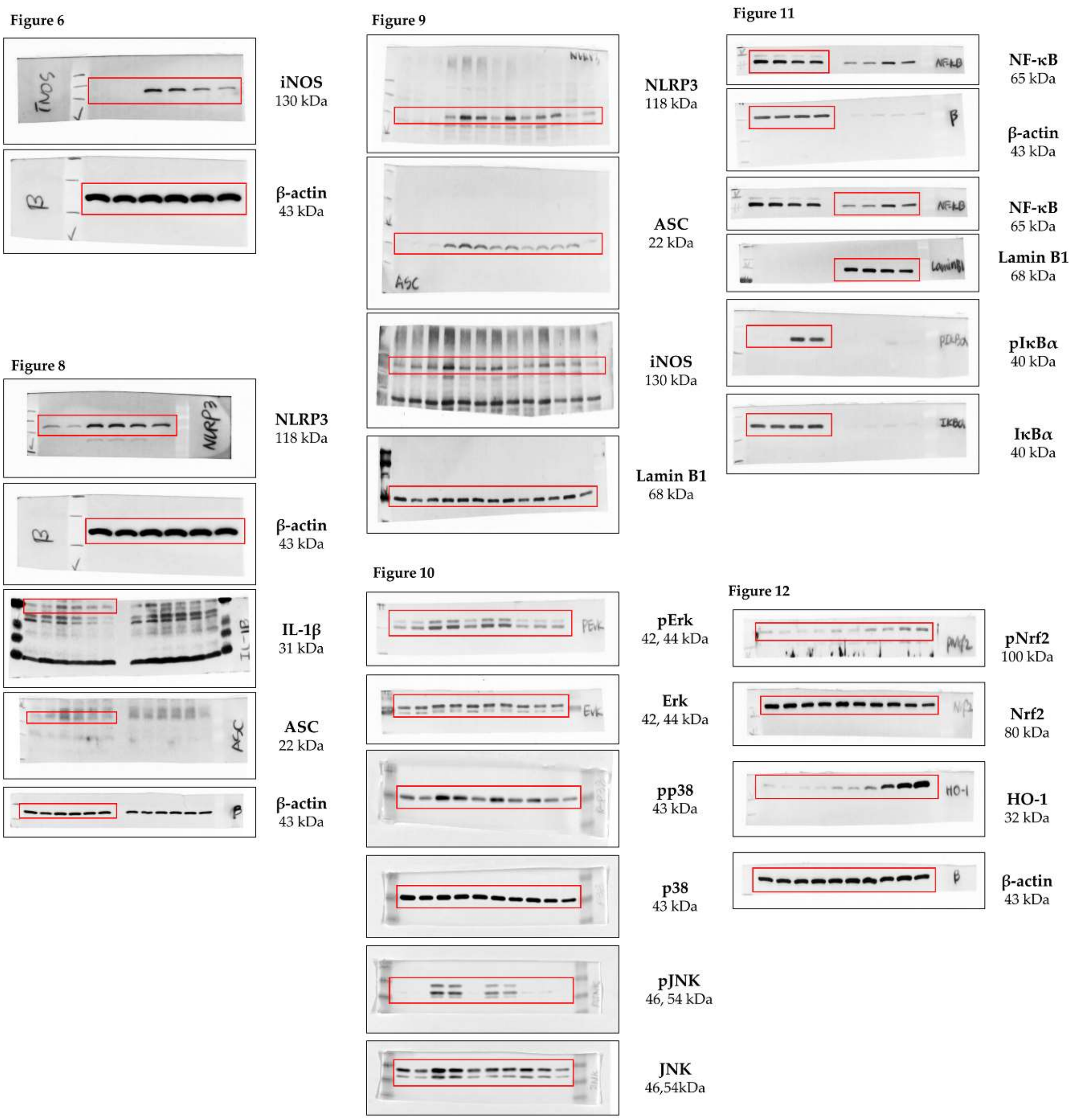

Figure S36. Full band for all western blot experiments
